# The Transcription Factors HbWRKY29 and HbPTI5 cooperatively enhance rubber tree resistance to powdery mildew

**DOI:** 10.1111/mpp.70293

**Published:** 2026-06-11

**Authors:** Yiying Lu, Qifeng Liu, Ling Xia, Suna Peng, Meng Wang, Xiaoyu Liang, Yu Zhang

**Affiliations:** ^1^ State Key Laboratory of Tropical Crop Breeding, Sanya Institute of Breeding and Multiplication, School of Tropical Agriculture and Forestry Hainan University Sanya China

**Keywords:** HbPTI5, HbWRKY29, powdery mildew resistance, rubber tree, transcription factors

## Abstract

Powdery mildew, caused by *Erysiphe quercicola*, is a major foliar disease that severely reduces the productivity of rubber tree ( *Hevea brasiliensis* ). Despite its economic importance, the transcriptional regulatory mechanisms underlying resistance to this pathogen remain unclear. In this study, two key transcription factors, HbWRKY29 and HbPTI5, were identified as cooperative regulators of defence responses against *E. quercicola* . Both HbWRKY29, a WRKY‐family member, and HbPTI5, an AP2/ERF‐type transcription factor, were rapidly induced during the early stages of infection. Yeast two‐hybrid (Y2H), bimolecular fluorescence complementation (BiFC), and luciferase complementation imaging (LCI) assays confirmed that HbWRKY29 physically interacts with HbPTI5 in the nucleus. HbWRKY29 also binds a W‐box element in the *HbPTI5* promoter, activating its transcription and forming a positive feedback loop to amplify immune signalling. Co‐expression of these two factors induced hypersensitive response (HR)‐like cell death and H_2_O_2_ accumulation, enhancing resistance to *E. quercicola* , whereas silencing either gene compromised defence responses. Furthermore, HbPTI5 acts as a transcriptional activator of *HbTLP1*, a thaumatin‐like protein belonging to the PR5 family with antifungal activity. Overexpression of *HbTLP1* enhanced resistance to powdery mildew, while silencing this gene weakened immunity. Collectively, these findings reveal a WRKY–ERF transcriptional cascade in rubber tree, which integrates rapid HR activation with sustained antifungal defence. This study provides new insights into the molecular mechanisms of rubber tree immunity, identifying potential targets for breeding disease‐resistant cultivars.

## Graphical abstract

Upon *Erysiphe quercicola* infection, rubber HbWRKY29 is rapidly induced and physically interacts with HbPTI5 to trigger HR. HbWRKY29 binds the *HbPTI5* promoter (W‐box), enhancing HbPTI5 expression, which activates HbTLP1 expression, leading to reinforced defence. This dual‐layered feedback cascade integrates immune activation with sustained resistance, conferring protection against powdery mildew.

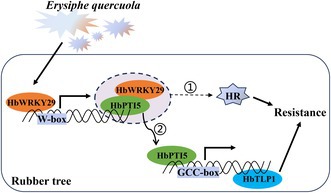

## 1 Introduction

Natural rubber, derived from *Hevea brasiliensis* , is a crucial industrial raw material used extensively in the automotive, aerospace, and medical sectors (Huang et al. 2024; Yang et al. 2025). As the largest global consumer of natural rubber, China faces significant challenges from rubber tree powdery mildew (*Erysiphe quercicola*), a prevalent foliar disease in plantations (Liyanage et al. 2016; Liu et al. 2024). The infection leads to leaf curling, necrosis, premature abscission, and in severe cases, reduced latex production or even tree death, resulting in substantial economic losses (Zhai et al. 2023; Cao et al. 2024). At present, the primary control measure for powdery mildew is the application of chemical fungicides, but these methods are costly and environmentally harmful (Arraño‐Salinas et al. 2018). As such, molecular breeding for disease‐resistant cultivars has emerged as a promising approach to managing this issue sustainably.

Plants possess a sophisticated immune system that can detect pathogen invasion and initiate defence responses. WRKY transcription factors (TFs) are key regulators in this system, controlling multiple defence pathways by specifically binding W‐box motifs in the promoters of target genes. Significant progress has been made in recent years in elucidating the roles of WRKY TFs in plant immunity. For example, studies on grapevine, tomato, and apple have shown that WRKYs are involved in resistance to powdery mildew (Yin et al. 2022; Wang, Wang, et al. 2023; Lan et al. 2025), rice blast and bacterial wilt (Hao et al. 2022), and grey mould (Huang et al. 2022). Certain WRKY transcription factors can rapidly trigger immune responses either through changes in their own expression levels or by forming regulatory complexes, such as WRKY–WRKY dimers, which act at transcriptional or post‐translational levels (Rushton et al. 2010; Dang et al. 2023; Zhou et al. 2024). Increasing evidence also indicates collaborative roles of WRKYs with other TF families in defence regulation. For instance, the AP2 TF CsAP2‐09 activates *CsWRKY25* to enhance citrus canker resistance (Li et al. 2024); JrWRKY4 interacts with the MYB TF *JrPHL8* to modulate walnut resistance to anthracnose (Mu et al. 2024); and *Arabidopsis* WRKYs form complexes with bZIP28 to regulate immune signalling under stress (Arraño‐Salinas et al. 2018). Research on WRKY TFs in rubber tree immunity is still limited, particularly regarding powdery mildew resistance. Although transcriptomic data indicate the activation of numerous TFs during pathogen challenge (Liang et al. 2023), the molecular mechanisms governing WRKY‐mediated resistance are yet to be fully understood.

In addition to WRKYs, the AP2/ERF family of TFs is crucial for regulating plant immune responses. In *Arabidopsis*, *AtERF14* positively regulates resistance to Fusarium wilt (Oñate‐Sánchez et al. 2007), and several ERF TFs contribute to rice blast resistance (Cao et al. 2006; Lin et al. 2007). ERF proteins also contribute to antiviral responses in tobacco (Cao et al. 2005). Recent studies have shown that ERF TFs often interact with other proteins to amplify immune responses. For example, in grapevine, VqERF062 forms a heterodimer with VqERF1B to enhance powdery mildew resistance (Yan et al. 2025). Similarly, the Pto–Pti signalling pathway in tomato involves ERF‐type TFs that interact with Pto kinase, triggering a hypersensitive response (HR) and activating pathogenesis‐related (PR) genes to strengthen disease resistance (Zhou et al. 1995; Zhou et al. 1997; Gu and Martin 1998; Gu et al. 2002). A recent study in walnut demonstrated that JrWRKY21 interacts with the ERF TF JrPTI5L to transcriptionally activate downstream PR genes, contributing to anthracnose resistance (Zhou et al. 2022). However, the role of ERF TFs in rubber tree immunity, particularly in relation to powdery mildew resistance, remains poorly understood.

In this study, we aimed to explore the functional interplay between the WRKY TF HbWRKY29 and the AP2/ERF TF HbPTI5 in rubber tree and their roles in powdery mildew resistance. We demonstrate that HbWRKY29 physically interacts with HbPTI5, activating its transcription and enhancing immune responses. Additionally, we show that HbPTI5 regulates the expression of HbTLP1, a thaumatin‐like protein that contributes to antifungal defence. Our findings provide valuable insights into the molecular mechanisms of powdery mildew resistance in rubber tree, offering potential targets for breeding disease‐resistant cultivars.

## 2 Results

### 2.1 HbWRKY29 and HbPTI5 Coordinate Early Responses to *E. quercicola* Infection in Rubber Tree

**FIGURE 1:**
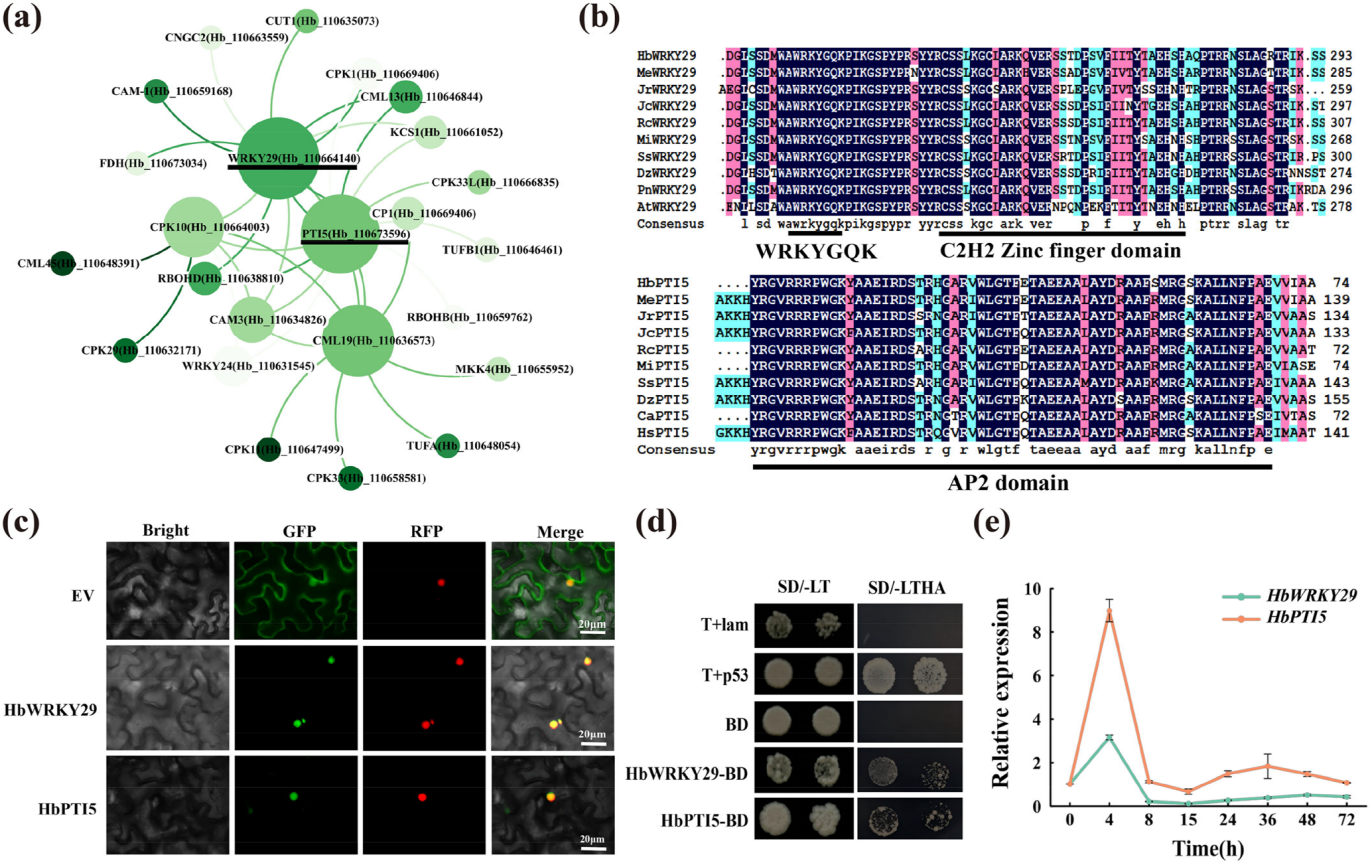
Response of *HbWRKY29* and *HbPTI5* to *Erysiphe quercicola* infection. (a) Gene co‐expression network of differentially expressed genes involved in plant–pathogen interactions in rubber tree during *E. quercicola* infection. Green dots represent gene nodes, and lines indicate expression correlations. (b) Alignment of partial amino acid sequences of WRKY29 and PTI5 with their homologous proteins in other plants. The conserved WRKY domain and AP2 domain are indicated by short horizontal lines. (c) Subcellular localization of HbWRKY29 and HbPTI5 proteins in *Nicotiana benthamiana*. Green signals indicate reconstituted GFP fluorescence, whereas red signals represent the RFP‐tagged nuclear marker 35S:H2B‐mCherry. (d) Yeast self‐activation assay for HbWRKY29 and HbPTI5. Self‐activation was tested on SD/−Trp/−Leu/−His/−Ade medium. (e) Temporal expression patterns of *HbWRKY29* and *HbPTI5* in rubber tree leaves following powdery mildew inoculation, with 0 h serving as the control.

Members of the WRKY transcription factor family are significantly upregulated in rubber tree leaf epidermal cells during infection by *E. quercicola* (Liang et al. 2023). Based on our previous single‐cell transcriptomic data, we constructed a plant–pathogen interaction network that revealed a strong correlation between *HbWRKY29* and *HbPTI5* expression (Figure 1a). Phylogenetic analysis indicated that HbWRKY29 is highly homologous to *Arabidopsis* AtWRKY29 and contains the conserved WRKYGQK motif and a C2H2 zinc‐finger domain (Figure 1b and Figure S1). HbPTI5 is an AP2/ERF‐family protein with a DNA‐binding AP2/ERF domain (Figure 1b), and both HbWRKY29 and HbPTI5 localize to the nucleus (Figure 1c). Furthermore, yeast transactivation assays confirmed that both proteins exhibit transcriptional activation activity (Figure 1d). Reverse transcription‐quantitative PCR (RT‐qPCR) analysis showed that both *HbWRKY29* and *HbPTI5* were significantly upregulated within 4 h post‐inoculation (hpi) with *E. quercicola* , suggesting early activation in response to pathogen invasion (Figure 1e). These findings indicate that HbWRKY29 and HbPTI5 act in concert to mediate the early immune response in rubber tree.

### 2.2 HbWRKY29 Interacts With HbPTI5 and Positively Regulates Its Expression

**FIGURE 2:**
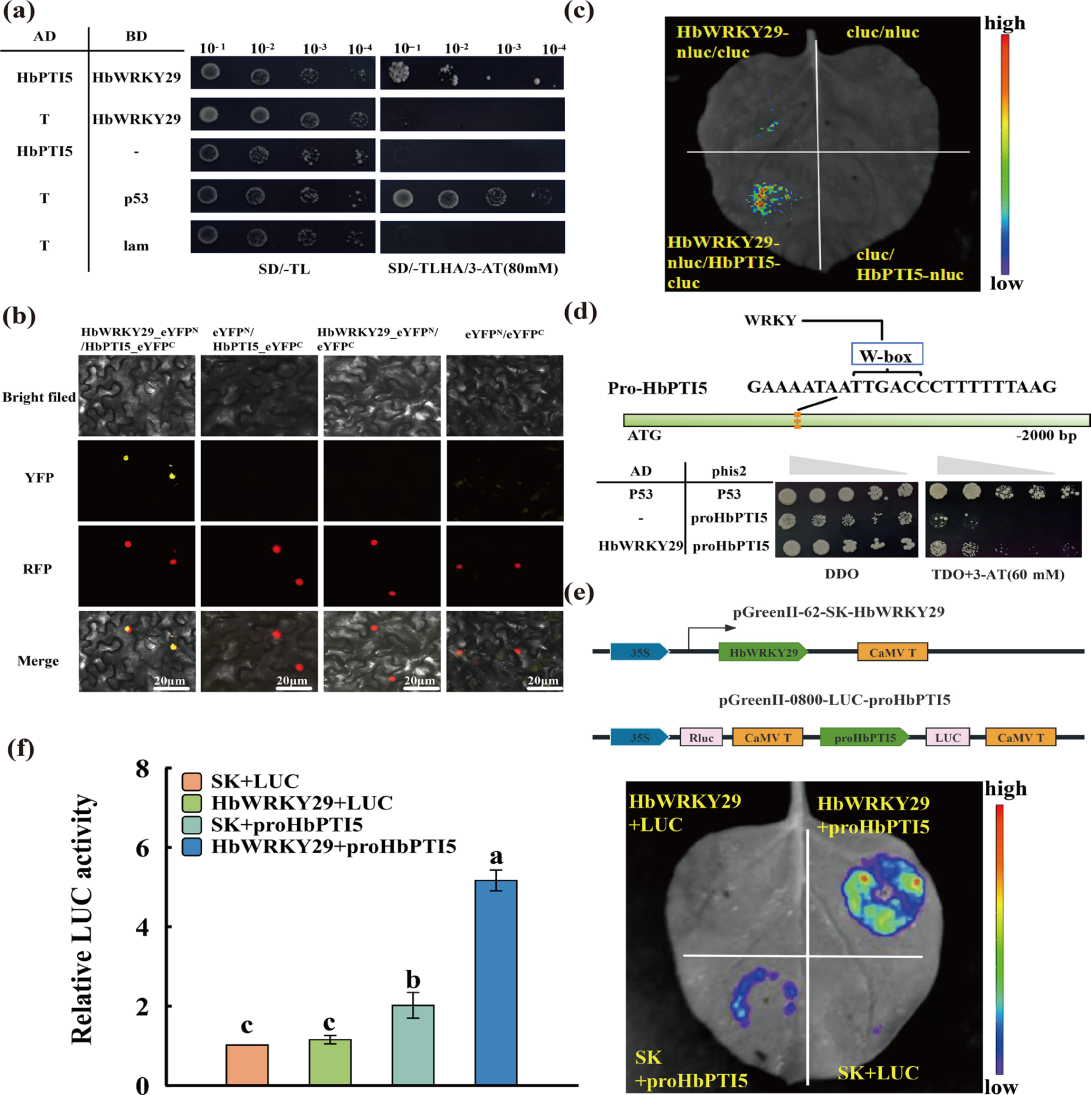
HbWRKY29 physically interacts with HbPTI5 and transcriptionally activates its expression. (a) Yeast two‐hybrid assay verifying the interaction. Yeast cells co‐transformed with plasmids were grown on SD/−Leu/−Trp/−His/−Ade medium. AD‐T/BD‐53 and AD‐T/BD‐lam served as positive and negative controls, respectively. (b) Bimolecular fluorescence complementation assay confirming the nuclear interaction in *Nicotiana benthamiana*. Yellow signals indicate reconstituted YFP fluorescence, whereas red signals represent the RFP‐tagged nuclear marker 35S:H2B‐mCherry. Scale bars, 20 μm. (c) Luciferase complementation imaging assay showing strong luminescence signals in leaves co‐expressing *HbWRKY29* and *HbPTI5*. (d) Yeast one‐hybrid assay demonstrating that HbWRKY29 binds to the *HbPTI5* promoter. (e) Schematic representation of the constructs used in the dual‐luciferase reporter (DLR) assay: pGreenII‐62‐SK‐HbWRKY29, an effector vector overexpressing HbWRKY29; and pGreenII‐0800‐LUC‐proHbPTI5, a reporter vector containing the HbPTI5 promoter driving LUC, with Renilla luciferase (Rluc) as an internal control. (f) DLR assay showing that HbWRKY29 significantly enhances *HbPTI5* promoter activity. The empty pGreenII‐62‐SK vector (SK) served as the negative control, while the promoterless pGreenII‐0800‐LUC vector (LUC) served as the reporter control. Different letters indicate statistically significant differences according to LSD test (*p* < 0.05).

To explore the relationship between HbWRKY29 and HbPTI5, we performed a yeast two‐hybrid (Y2H) assay, which confirmed that the two proteins physically interact (Figure 2a). Bimolecular fluorescence complementation (BiFC) and firefly luciferase complementation imaging (LCI) assays further demonstrated that HbWRKY29 and HbPTI5 proteins interact in the nucleus (Figure 2b,c). Analysis of the *HbPTI5* promoter sequence identified a W‐box element approximately 743 bp upstream of the transcription start site, suggesting a potential WRKY‐binding site. Yeast one‐hybrid (Y1H) assays showed that HbWRKY29 binds the *HbPTI5* promoter fragment containing this W‐box (Figure 2d). Moreover, a dual‐luciferase reporter (DLR) assay demonstrated that HbWRKY29 significantly enhanced transcriptional activity from the *HbPTI5* promoter (Figure 2e,f). Together, these results indicate that HbWRKY29 directly binds the *HbPTI5* promoter and activates HbPTI5 expression.

### 2.3 Co‐Expression of *HbWRKY29* and *HbPTI5* Induces HR and Suppresses Pathogen Infection

**FIGURE 3:**
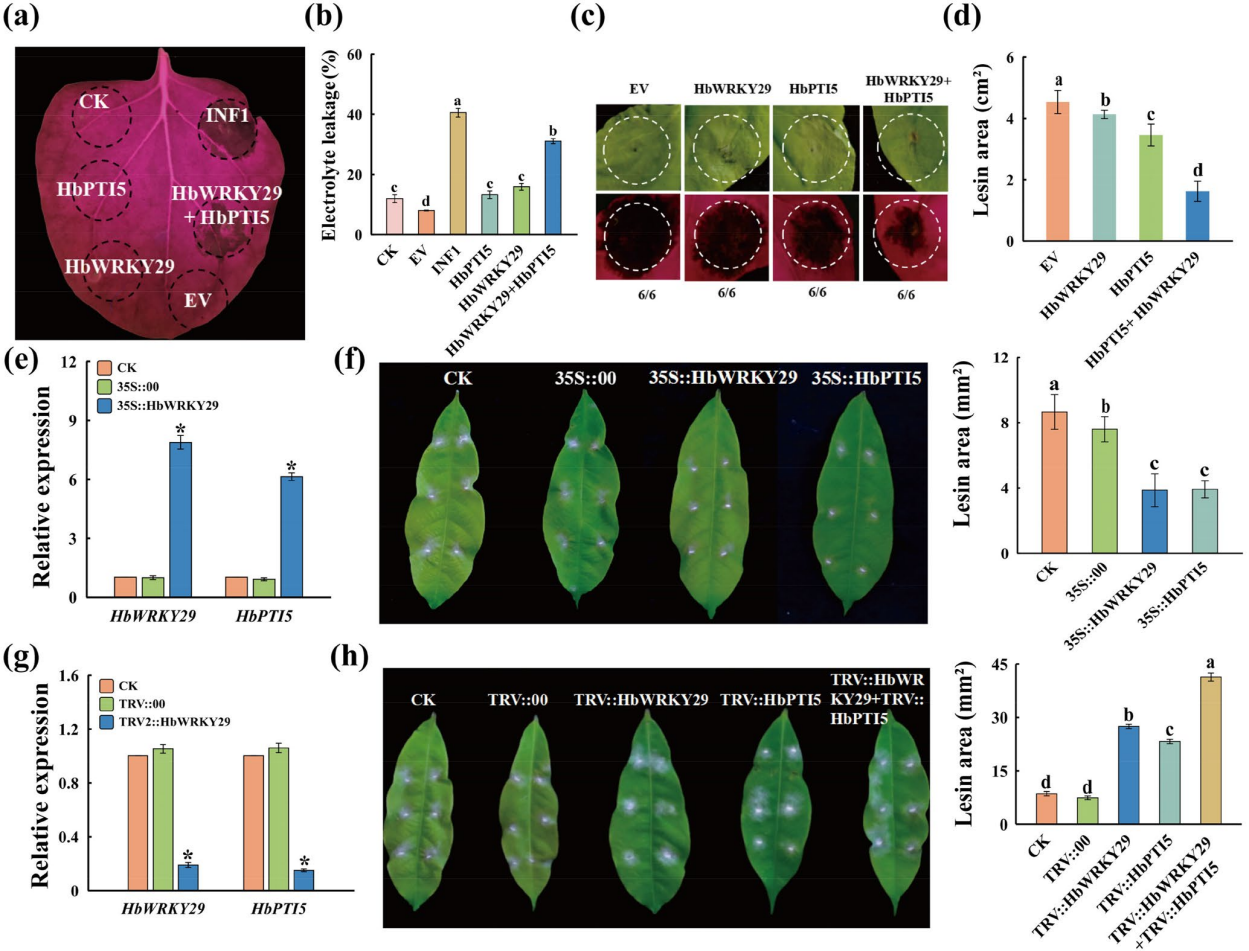
Co‐expression of *HbWRKY29* and *HbPTI5* triggers hypersensitive response (HR) and enhances resistance to pathogen infection. (a, b) Co‐expression of *HbWRKY29* and *HbPTI5* inducing visible cell death and increasing electrolyte leakage compared with single‐gene expression. CK, buffer control; EV, empty vector. (c) Co‐expression of *HbWRKY29* and *HbPTI5* enhances resistance to *Phytophthora nicotianae* and reduces lesion area. Leaves were photographed under normal light (upper panel) and ultraviolet light (lower panel). Leaves were photographed under white light and ultraviolet light at 48 h after agroinfiltration for HR observation. *P. nicotianae* was inoculated at 24 h after agroinfiltration, and disease phenotypes were recorded at 48 h post‐inoculation. “6/6” indicates that six out of six tested leaves displayed similar phenotypes. Lesion areas are shown in (d). (e) Relative expression levels of *HbWRKY29* and *HbPTI5* in transiently overexpressed in rubber tree leaves compared with CK (non‐overexpressing leaves). (f) Disease symptoms and lesion areas in *HbWRKY29*‐ and *HbPTI5*‐overexpressing rubber tree leaves following *Erysiphe quercicola* infection. (g) Relative expression levels of *HbWRKY29* and *HbPTI5* in rubber tree leaves under virus‐induced gene silencing treatment compared with control (CK; non‐silenced leaves). (h) Disease symptoms and lesion areas in *HbWRKY29*‐ and *HbPTI5*‐silenced rubber tree leaves following *E. quercicola* infection. Different letters indicate statistically significant differences according to the LSD test (*p < 0.05).

To examine the immune function of *HbWRKY29* and *HbPTI5*, we conducted transient overexpression in *Nicotiana benthamiana* leaves. Expression of either *HbWRKY29* or *HbPTI5* alone did not trigger HR; however, co‐expression of both genes induced clear programmed cell death and increased electrolyte leakage in *N. benthamiana* leaves (Figure 3a,b). Consistently, 3,3′‐diaminobenzidine (DAB) staining showed significantly higher H_2_O_2_ accumulation in leaves co‐expressing *HbWRKY29* and *HbPTI5* than in leaves expressing either gene alone (Figure S2), indicating an intensified oxidative burst. This suggests that the combined action of *HbWRKY29* and *HbPTI5* triggers HR. To test whether this translates into increased disease resistance, we inoculated *N. benthamiana* leaves co‐expressing *HbWRKY29* and *HbPTI5* with *Phytophthora nicotianae*. These leaves developed significantly smaller lesions than leaves expressing either gene alone (Figure 3c), indicating that the synergistic action of *HbWRKY29* and *HbPTI5* triggers HR and enhances disease resistance.

### 2.4 *HbWRKY29* and *HbPTI5* Positively Regulate Rubber Tree Resistance to Powdery Mildew

We then investigated the roles of *HbWRKY29* an*d HbPTI5* in rubber tree using transient overexpression and virus‐induced gene silencing (VIGS) assays. In transient overexpression experiments on seedling leaves, *HbWRKY29* overexpression significantly upregulated transcripts of both *HbWRKY29* and *HbPTI5* (Figure 3d). Upon *E. quercicola* inoculation, leaves overexpressing *HbWRKY29* developed markedly smaller powdery mildew colonies after 7 days compared to control leaves (Figure 3e), indicating that *HbWRKY29* enhances powdery mildew resistance. Similarly, overexpression of *HbPTI5* increased *HbPTI5* expression (Figure S3a) and markedly reduced disease symptoms at 7 days post‐inoculation (dpi) (Figure 3e).

In gene silencing experiments, silencing *HbWRKY29* led to significantly decreased expression of both *HbWRKY29* and *HbPTI5* (Figure 3f). Leaves in which either *HbWRKY29* or *HbPTI5* were silenced exhibited enlarged lesions relative to controls (Figure 3g and Figure S3b), indicating a compromised defence response. Moreover, co‐silencing both genes resulted in even more severe disease symptoms than single‐gene silencing (Figure 3g and Figure S4), further supporting a synergistic, positive regulatory relationship between *HbWRKY29* and *HbPTI5* in mediating powdery mildew resistance.

### 2.5 HbPTI5 Acts as a Transcriptional Activator of *HbTLP1*

**FIGURE 4:**
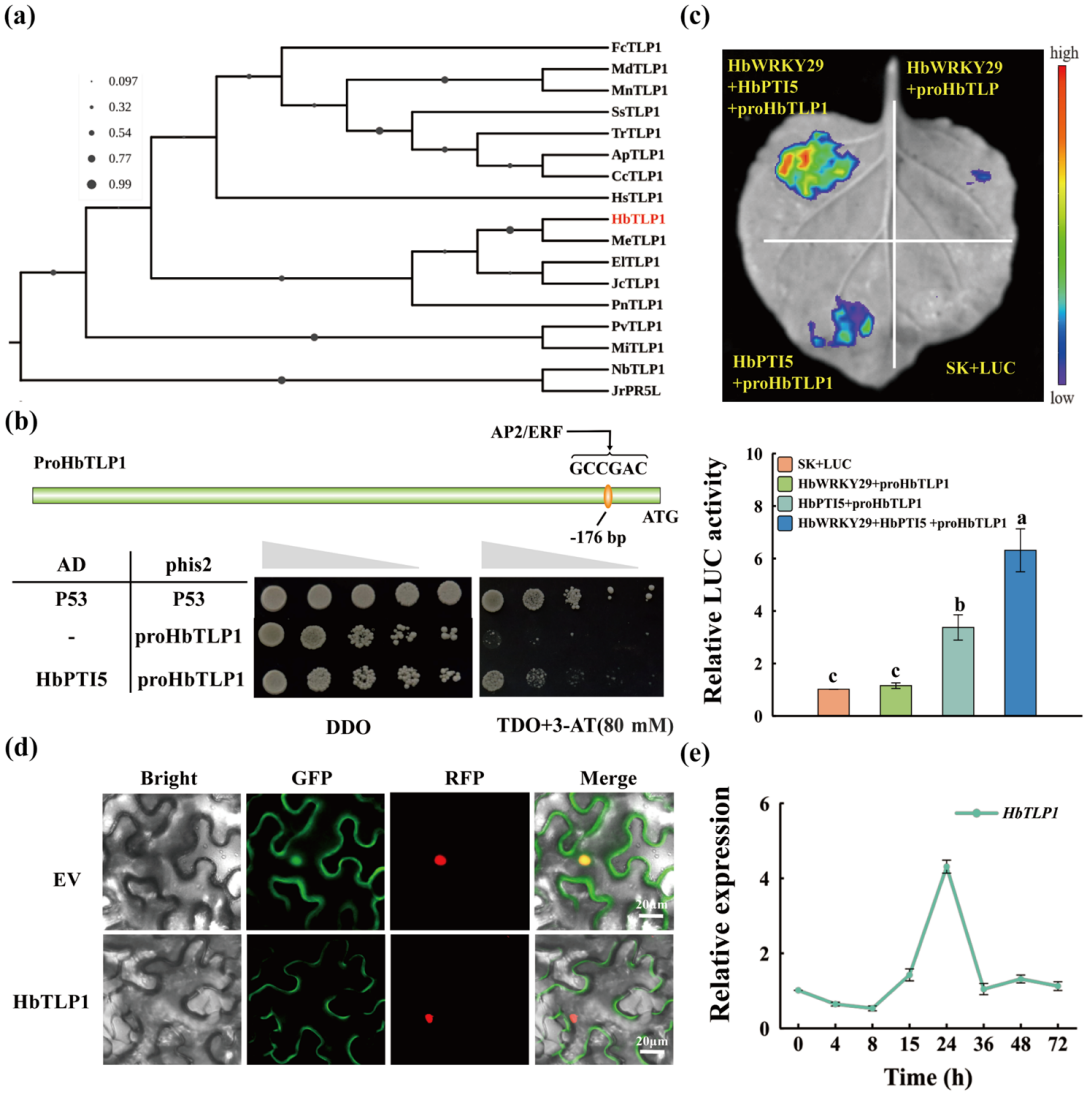
HbPTI5 acts as a transcriptional activator of *HbTLP1*. (a) Phylogenetic analysis of TLP1 proteins from rubber tree and representative plant species. (b) Diagram of *HbTLP1* promoter region. Yeast one‐hybrid assay demonstrating the binding of HbPTI5 to the *HbTLP1* promoter. (c) Dual‐luciferase reporter assay showing that HbPTI5 significantly activates *HbTLP1* transcriptional activity. (d) Subcellular localization of HbTLP1 in *Nicotiana benthamiana* leaves. Green signals indicate reconstituted GFP fluorescence, whereas red signals represent the RFP‐tagged nuclear marker 35S:H2B‐mCherry. Scale bars, 20 μm. (e) Temporal expression patterns of *HbTLP1* in rubber tree leaves following powdery mildew inoculation, with 0 h serving as the control. Different letters indicate statistically significant differences according to the LSD test (*p* < 0.05).

In plant immune responses, AP2/ERF family transcription factors often play key roles by activating the expression of pathogenesis‐related (PR) genes. Thaumatin‐like protein 1 (TLP1) is an important PR protein involved in plant resistance to pathogens. Phylogenetic analysis showed that HbTLP1 is a thaumatin‐like protein with low sequence homology to walnut JrPR5L (Figure 4a), which is known to be regulated by JrPTI5. To determine whether HbPTI5 functions as a transcriptional activator of *HbTLP1*, we analysed the *HbTLP1* promoter and identified a GCCGAC motif (an ERF‐binding element) about 176 bp upstream of the transcription start site. Yeast one‐hybrid (Y1H) assays confirmed that HbPTI5 can bind to the *HbTLP1* promoter, and dual‐luciferase reporter assays demonstrated that HbPTI5 significantly enhanced *HbTLP1* transcriptional activity (Figure 4b,c). The open reading frame of *HbTLP1* encodes a protein of 298 amino acids, with a predicted signal peptide located at positions 1–25 (Figure S5a). SignalP analysis predicted a clear signal peptide cleavage site between residues 25 and 26, suggesting that HbTLP1 is probably a secreted protein (Figure S5b). Hydrophobicity analysis showed that the N‐terminal region exhibits strong hydrophobicity, whereas most regions of the mature protein are hydrophilic (Figure S5c). In addition, transmembrane topology prediction identified a putative transmembrane region within the signal peptide sequence at the N‐terminus, while the remaining protein was predicted to be mainly extracellular (Figure S5d). Subcellular localization analysis showed that HbTLP1‐GFP fluorescence was predominantly detected at the cell periphery, consistent with the predicted secretory characteristics of HbTLP1 (Figure 4d). Furthermore, reverse transcription‐quantitative PCR (RT‐qPCR) analysis revealed that the expression of *HbTLP1* increased significantly (approximately 3.8‐fold) within 24 h after *E. quercicola* infection (Figure 4e), supporting an important role for *HbTLP1* in the rubber tree immune response.

### 2.6 *HbTLP1* Positively Regulates Rubber Tree Resistance to Powdery Mildew

**FIGURE 5:**
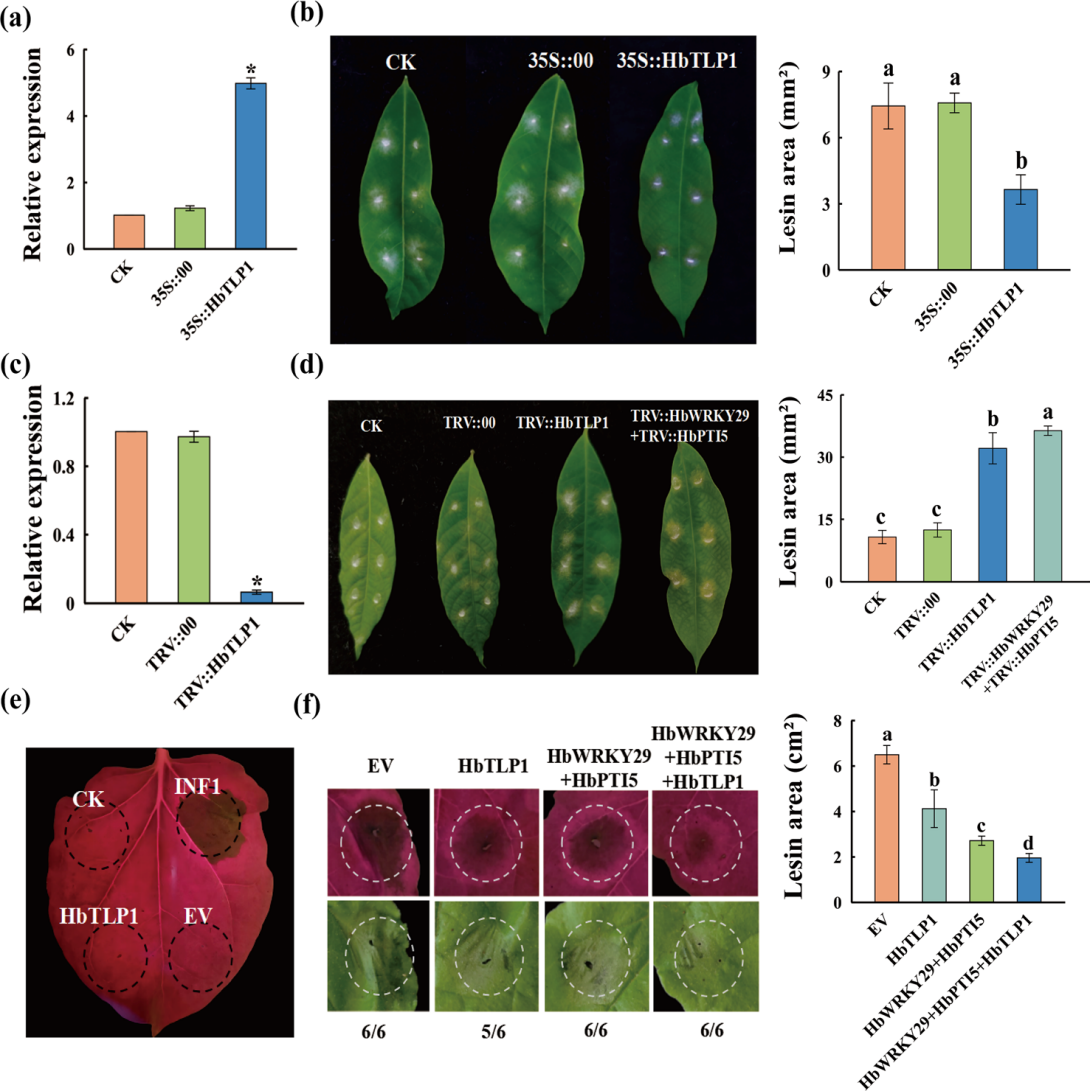
*HbTLP1* positively regulates rubber tree resistance to powdery mildew. (a) Relative expression levels of *HbTLP1* when transiently overexpressed in rubber tree leaves compared with control (CK). (b) Disease symptoms and lesion areas in *HbTLP1*‐overexpressing rubber tree leaves after *Erysiphe quercicola* infection. (c) Relative expression levels of *HbTLP1* in virus‐induced gene silencing‐treated leaves compared with CK. (d) Disease symptoms and lesion areas in *HbTLP1*‐silenced rubber tree leaves after infection. (e) Transient expression of *HbTLP1* in *Nicotiana benthamiana* leaves does not trigger hypersensitive response‐like cell death. (f) Co‐expression of *HbWRKY29*, *HbPTI5*, and *HbTLP1* reduced lesion areas after *Phytophthora nicotianae* infection. Different letters indicate statistically significant differences according to the LSD test (*p* < 0.05).

To verify the role of *HbTLP1* in rubber tree powdery mildew resistance, we transiently overexpressed *HbTLP1* in rubber tree leaves (Figure 5a). Overexpression of *HbTLP1* markedly enhanced rubber tree resistance to *E. quercicola* , as evidenced by significantly smaller lesions on leaves at 7 dpi compared to controls (Figure 5b). Conversely, VIGS‐mediated silencing of *HbTLP1* substantially reduced powdery mildew resistance, with silenced leaves showing much larger lesions (Figure 5c,d). Moreover, co‐silencing *HbWRKY29* and *HbPTI5* in rubber tree leaves led to a notably lower expression of *HbTLP1* than silencing *HbPTI5* alone (Figure S6). These results indicate that the synergistic action of HbWRKY29 and HbPTI5 is required to fully activate HbTLP1, thereby strengthening the rubber tree immune response to powdery mildew. Additionally, we explored the contribution of *HbTLP1* to pathogen resistance in *N. benthamiana* leaves. Transient overexpression of *HbTLP1* alone did not induce HR (Figure 5e), but upon *P. nicotianae* inoculation, lesion spread was limited in *HbTLP1*‐expressing leaves. When *HbWRKY29*, *HbPTI5*, and *HbTLP1* were co‐expressed, the lesion size further decreased (Figure 5f). This finding further supports that HbWRKY29 and HbPTI5 enhance disease resistance by activating *HbTLP1* expression.

## 3 Discussion

WRKY TFs are central regulators of plant immunity and are widely implicated in defence against powdery mildew pathogens across diverse species. For example, WRKY‐based regulatory modules activate canonical defence responses, including jasmonate‐associated signalling in *Arabidopsis*, systemic acquired resistance in apple (Pandey et al. 2010), and callose deposition with hypersensitive cell death in grapevine (Zhang et al. 2025). These findings underscore the conserved importance of WRKY TFs in coordinating immune transcriptional reprogramming during fungal invasion. By contrast, the contribution of WRKY TFs to the immune system of the rubber tree has remained largely unexplored.

In this study, we identified HbWRKY29 as a rapidly inducible defence‐associated WRKY in rubber tree that responds strongly to *E. quercicola* infection. The temporal expression pattern of HbWRKY29 was tightly synchronized with that of the AP2/ERF TF HbPTI5 during the early stages of pathogen challenge, suggesting a cooperative role for these two TFs in rubber tree immunity. Co‐expression of *HbWRKY29* and *HbPTI5* induces a strong HR, whereas expression of either TF alone results in a weak or no detectable defence response. Consistent with these transient assays, overexpression of *HbWRKY29* or *HbPTI5* in rubber tree enhanced resistance to powdery mildew, whereas silencing either gene compromised resistance, with the greatest susceptibility observed when both genes were co‐silenced. Together, these observations support the conclusion that HbWRKY29 and HbPTI5 do not function independently but instead act synergistically to initiate a robust immune response against *E. quercicola* .

Although WRKY–ERF regulatory interactions have been reported in other woody perennials, such as walnut, citrus, and apple (Liu et al. 2023; Li et al. 2024), such cooperative interactions in rubber tree had not been documented before this study. Our study therefore provides the first evidence of a WRKY–ERF immune regulatory module in rubber tree, expanding understanding of transcriptional regulation in this important crop. This discovery not only sheds light on the molecular mechanisms governing powdery mildew resistance but also suggests that WRKY–ERF cooperative signalling is an evolutionarily conserved strategy in perennial species repeatedly exposed to pathogen pressures throughout their long lifespans.

**FIGURE 6:**
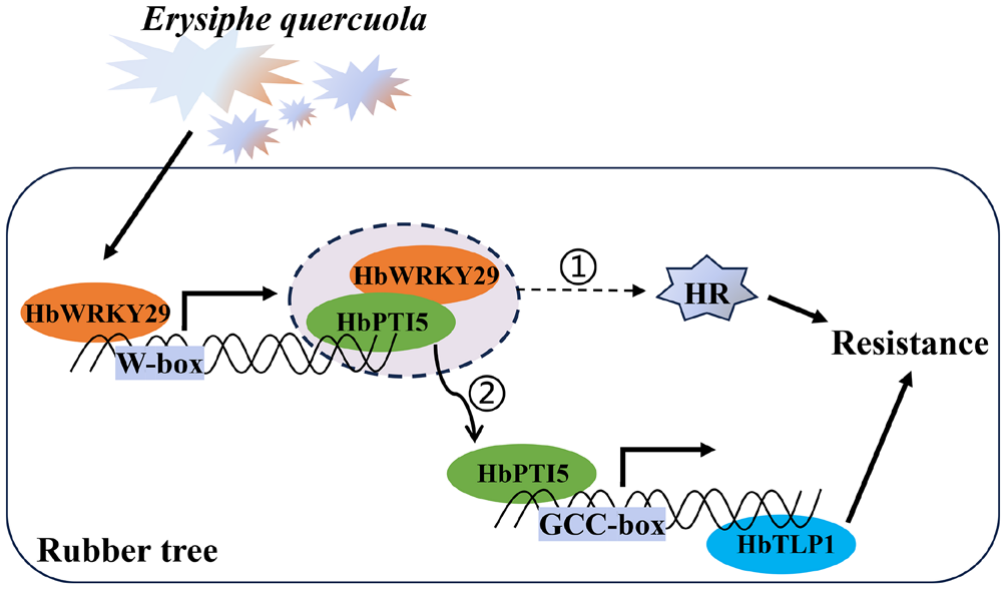
Proposed model of the HbWRKY29–HbPTI5 regulatory cascade in rubber tree defence against *Erysiphe quercicola*. Upon *E. quercicola* infection, HbWRKY29 is rapidly induced and physically interacts with HbPTI5 to trigger hypersensitive response (HR). HbWRKY29 also binds the *HbPTI5* promoter (W‐box), enhancing HbPTI5 expression and forming a positive regulatory loop. HbPTI5 subsequently activates HbTLP1 expression by recognizing the GCC‐box motif, leading to reinforced antifungal defence. This dual‐layered feedback cascade integrates rapid immune activation with sustained resistance, conferring effective protection against powdery mildew.

Mechanistically, the HbWRKY29–HbPTI5 module operates as a dual‐layer defence mechanism that integrates both rapid immune activation and sustained antifungal protection (Figure 6). At early infection stages, the physical interaction between HbWRKY29 and HbPTI5 triggers HR, providing an immediate barrier to pathogen invasion. At the same time, HbWRKY29 directly binds the W‐box element in the *HbPTI5* promoter and enhances *HbPTI5* transcription, thus establishing a positive feedback loop that ensures the accumulation of sufficient HbPTI5 to relay immune signals. Members of the PTI protein family, including PTI4, PTI5 and PTI6, possess the typical characteristics of transcription factors. Their functions are comparable to those of ethylene‐responsive element binding proteins (EREBPs) identified in tobacco by Ohme‐Takagi and Shinshi (1995), which specifically recognize the GCC‐box *cis*‐acting element (GCCGAC). In tomato (Ohme‐Takagi and Shinshi 1995), PTI proteins bind directly to GCC‐box motifs in PR gene promoters and activate their transcription through distinct regulatory mechanisms (Zhou et al. 1997; Gu et al. 2000). Collectively, these studies demonstrate that PTI family proteins function as positive regulators of plant defence and represent key components of the transcriptional network governing immune responses. Consistent with these findings, HbPTI5 in rubber tree probably functions as a downstream effector‐type TF that activates the expression of defence‐related genes such as *HbTLP1* by binding to GCC‐box motifs within their promoters. Overexpression of *TaTLP1* has been shown to significantly enhance wheat resistance to leaf rust caused by *Puccinia triticina* and root rot induced by *Bipolaris sorokiniana* (Cui et al. 2021). Furthermore, TaTLP1 interacts with TaPR1 to promote wheat's resistance to *P. triticina* (Wang et al. 2020). Increased expression of TLP proteins enhances both structural and biochemical defences, limiting fungal colonization and disease spread (van Loon et al. 2006; de Jesús‐Pires et al. 2020). Thus, the HbWRKY29–HbPTI5 module functions as a central signalling hub, coordinating rapid pathogen containment with long‐lasting antifungal defence—crucial for perennial species like rubber tree that must contend with repeated pathogen exposures over decades.

In conclusion, this study supports a model in which the HbWRKY29–HbPTI5 cascade serves as a key regulatory module in the powdery mildew resistance pathway of rubber trees, coordinating rapid pathogen containment with durable antifungal defence, a feature particularly important for perennial plants such as rubber trees, which must cope with repeated infections over several decades. By integrating single‐cell transcriptomics with molecular, genetic, and physiological analyses, this study provides a comprehensive framework for understanding how TF modules shape immune outputs in rubber trees. These results lay a molecular foundation for breeding rubber tree cultivars with enhanced resistance to powdery mildew and may inform strategies to engineer similar WRKY–ERF–PR modules in other economically important crops to improve disease resistance and support sustainable production.

## 4 Experimental Procedures

### 4.1 Plant Materials and Growth Conditions

Rubber tree seedlings (*Hevea brasiliensis*, 2–3 months old) were grown in a greenhouse at 25°C–28°C under a 16 h light/8 h dark photoperiod with 70% relative humidity. *N. benthamiana*, used for transient assays, were cultivated under similar conditions for 4–6 weeks. Healthy fully expanded leaves (3rd–5th from the apex) were selected for pathogen inoculation and agroinfiltration experiments.

### 4.2 Pathogen Strains and Inoculation

The powdery mildew fungus *E. quercicola* was maintained on rubber tree leaves at 22°C. 2 μL of the prepared spore suspension (10^5^ spores mL^−1^ in 0.02% Tween 80) was spotted onto the leaves of silenced or overexpressing rubber tree plants using a micropipette. Each leaf was inoculated with 6–8 spots, and three plants were inoculated. Inoculated plants were kept in a moist chamber at 22°C. The inoculation method for VIGS involves applying the prepared spore suspension to silenced rubber tree leaves using quantitative dot inoculation. Six inoculation points are applied per leaf, and three silenced plants are inoculated. For *P. nicotianae* , the isolate was grown on V8 agar for 10 days at 28°C. Fungal disks were punched from the outer edge of the colony and inoculated onto moistened *N. benthamiana* leaves. After 24 h of dark incubation under humid conditions, the disks were removed and cultured under normal light for 48 h. Disease symptoms were observed under both normal and ultraviolet light.

### 4.3 Gene Cloning and Phylogenetic Analysis

Total RNA was extracted from rubber tree leaves using RNAprep Pure Polysaccharide‐Polyphenol Plant Total RNA Kit (Tiangen) following the manufacturer's protocol. First‐strand cDNA was synthesized using PrimeScript RT kit (TaKaRa). Full‐length coding sequences (CDSs) of *HbWRKY29*, *HbPTI5*, and *HbTLP1* were amplified by PCR with gene‐specific primers (Table S1) using Phusion high‐fidelity DNA polymerase (NEB). PCR products were cloned into pGEM‐T Easy (Promega) or a similar entry vector and confirmed by Sanger sequencing. Homologous WRKY and AP2/ERF protein sequences from other plant species were retrieved from GenBank and aligned with ClustalW, with their GenBank accession numbers provided in Table S2. A phylogenetic tree was constructed by the neighbour‐joining method in MEGA X with 1000 bootstrap replicates to confirm orthology.

### 4.4 Vector Construction and *Agrobacterium* Infiltration

For subcellular localization and protein interaction assays, HbWRKY29, HbPTI5, and HbTLP1 coding sequences (without stop codons) were fused in‐frame to fluorescent or luciferase tags. Specifically, CDS fragments were cloned into binary vectors such as pCAMBIA1300‐35S::GFP, pSPYNE‐35S (eYFP^N^), pSPYCE‐35S (eYFP^C^), and split luciferase vectors (pCAMBIA‐nLUC, pCAMBIA‐cLUC), using restriction digestion or Gateway cloning. For Y2H assays, CDSs were inserted into pGADT7 (AD) and pGBKT7 (BD) vectors (Clontech). For Y1H assays, CDSs were inserted into pGADT7 (AD), and the HbPTI5, HbTLP1 promoter (~2.0 kb upstream of ATG) was cloned into pHis2. For dual‐luciferase reporter assays, the HbPTI5 promoter (~2.0 kb upstream of ATG) was cloned into pGreenII‐0800‐LUC, and HbWRKY29 CDS was cloned into pGreenII‐62‐SK. For the transient overexpression experiment, the CDS was inserted into pBin‐308‐myc. For VIGS, a 300 bp fragment of the CDS region was inserted into pYL156 (TRV RNA2). Plasmid constructs were transformed into *Agrobacterium tumefaciens* GV3101 by electroporation. All primers in this study were listed in Table S1.

*Agrobacterium* cultures were grown overnight in Luria Bertani (LB) medium with appropriate antibiotics at 28°C, collected by centrifugation, and resuspended in infiltration buffer (10 mM MgCl_2_, 10 mM MES pH 5.6, 100 μM acetosyringone) to an OD_600_ = 0.6. Suspensions were incubated at room temperature for 2 h before infiltration. For co‐expression assays, *Agrobacterium* cultures carrying different constructs were mixed at a 1:1 ratio by volume unless stated otherwise.

### 4.5 Subcellular Localization

Subcellular localization of HbWRKY29 and HbPTI5 was examined in *N. benthamiana* leaves. *Agrobacterium* carrying HbWRKY29::GFP or HbPTI5::GFP was infiltrated into the leaf underside. After 48 h, GFP fluorescence was observed under an Olympus BX53 upright fluorescence microscope with excitation at 488 nm and emission at 500–550 nm. The nuclear localization marker 35S:H2B‐mCherry was detected as RFP fluorescence (excitation: 558 nm; emission: 610–670 nm). Three biological replicates were performed per treatment, with three leaves examined per replicate and three cells observed per leaf.

### 4.6 Y2H Assay

Protein–protein interaction between HbWRKY29 and HbPTI5 was tested by Y2H. The HbWRKY29 CDS was cloned into pGBKT7 (binding domain, BD), and HbPTI5 into pGADT7 (activation domain, AD), and vice versa for reciprocal constructs. The BD and AD plasmids were co‐transformed into *Saccharomyces cerevisiae* AH109 using the polyethylene glycol/lithium acetate method. Transformants were selected on synthetic dropout (SD) medium lacking leucine and tryptophan (SD−Leu−Trp). Positive colonies were restreaked on SD medium lacking Leu, Trp, His, and Ade (SD−Leu−Trp−His−Ade) with 3‐amino‐1,2,4‐triazole (3‐AT) to suppress autoactivation. Yeast plates were incubated at 28°C for 3–5 days, and growth was scored (Wang, Xiao, et al. 2023).

### 4.7 BiFC Assay

BiFC was used to confirm interaction in planta. The HbWRKY29 and HbPTI5 CDS were fused to the N‐terminal or C‐terminal halves of YFP in pSPYNE‐35S and pSPYCE‐35S vectors. *Agrobacterium* strains carrying HbWRKY29‐eYFP^N^ and HbPTI5‐eYFP^C^ constructs (and the reciprocal fusion pairs) were co‐infiltrated into *N. benthamiana* leaves. After 48–72 h, YFP fluorescence indicating interaction was detected with excitation at 514 nm and emission at 525–575 nm. RFP fluorescence from the nuclear marker 35S:H2B‐mCherry was detected using excitation at 558 nm and emission at 610–670 nm. Controls included co‐expression of each fusion with the corresponding empty YFP half. Three biological replicates were performed for each treatment, with more than three cells observed per replicate.

### 4.8 LCI Assay

Protein interaction was further tested by LCI. The CDS of HbWRKY29 was fused to the N‐terminus of luciferase (nLUC) in pCAMBIA‐nLUC, and the HbPTI5 fused to the C‐terminus of luciferase (cLUC) in pCAMBIA‐cLUC. *Agrobacterium* carrying nLUC and cLUC fusions was mixed 1:1 and infiltrated into *N. benthamiana* leaves. After 48–72 h, infiltrated areas were sprayed with 1 mM D‐luciferin and imaged with in Vivo Imaging Analysis System. Luminescence signals were recorded and compared to negative controls (empty vectors). Each treatment was repeated three times. Three biological replicates were analysed for each treatment. Each biological replicate included three leaves.

### 4.9 Y1H Assay

To test DNA–protein interaction, a fragment of the *HbPTI5* promoter containing the putative W‐box motif (~743 bp upstream of ATG) was cloned into the pHis2 reporter vector. The HbWRKY29 CDS was cloned into pGADT7 (AD). The pHis2‐HbPTI5pro and pGADT7‐HbWRKY29 plasmids were co‐transformed into yeast strain Y187. Transformants were selected on SD medium lacking Trp, His and Leu (SD/−Trp−His−Leu). Interaction was assessed by growth on SD/−Trp−His−Leu medium supplemented with 3‐AT (determined empirically, typically 10 mM) to suppress background. Growth at 28°C for 3–5 days indicated binding of HbWRKY29 to the promoter fragment.

### 4.10 DLR Assay

Transient transcriptional activation was measured using a dual‐luciferase reporter system. The *HbPTI5* promoter (~2000 bp) was cloned upstream of the firefly luciferase (LUC) gene in pGreenII‐0800‐LUC; a Renilla luciferase (REN) under a constitutive promoter served as internal control. The *HbWRKY29* CDS was inserted into pGreenII‐62‐SK to express *HbWRKY29* as effector. *Agrobacterium* cultures carrying effector and reporter constructs (in a 10:1 volume ratio) were co‐infiltrated into *N. benthamiana* leaves. After 48 h, leaf disks were collected, and LUC and REN activities were measured using a dual‐luciferase assay kit (Promega) on a luminometer. The ratio of LUC to REN was calculated for each sample. Empty vector controls (no effector) were used to determine basal promoter activity. Three biological replicates were analysed for each treatment. Each biological replicate included three leaves.

### 4.11 Bioinformatic Analysis of HbTLP1

The amino acid sequence of HbTLP1 was analysed for conserved domains using the NCBI Conserved Domain Database. Signal peptide prediction was conducted using SignalP 5.0 to determine the presence and cleavage site of the N‐terminal signal peptide (Almagro Armenteros et al. 2019). Protein hydrophobicity profiles were analysed using the ProtScale program based on the Kyte–Doolittle algorithm (Kyte and Doolittle 1982). Transmembrane topology and intracellular/extracellular localization probabilities were predicted using the DeepTMHMM v. 1.0.24 servers (Hallgren et al. 2022).

### 4.12 VIGS in Rubber Tree

Gene silencing in rubber tree was performed using tobacco rattle virus (TRV)‐based VIGS. Gene‐specific fragments (~300 bp) of *HbWRKY29*, *HbPTI5* and *HbTLP1* were PCR‐amplified (primers in Table S1) and inserted into the pTRV2 vector. pTRV2 empty vector served as a negative control. *A. tumefaciens* GV3101 strains carrying pTRV1 and each pTRV2 construct were grown, resuspended in infiltration buffer to OD_600_ = 1.0, and incubated for 4 h. Equal volumes of TRV1 and TRV2 suspensions were mixed and infiltrated into the fully expanded leaves of 2‐week‐old rubber tree seedlings using a needleless syringe. Infiltrated plants were maintained at 22°C under a 12 h light/12 h dark. After 2 weeks, silencing efficiency was verified by RT‐qPCR, and plants were used for *E. quercicola* inoculation and phenotype observation. Three biological replicates were analysed for each treatment. Each biological replicate included six leaves.

### 4.13 Transient Expression and HR Assay

Transient overexpression of *HbWRKY29*, *HbPTI5* and *HbTLP1* was performed by *Agrobacterium*‐mediated infiltration. For *N. benthamiana* HR assays, *A. tumefaciens* GV3101 carrying HbWRKY29‐pBin, HbPTI5‐pBin, or a pBin‐myc control were infiltrated into *N. benthamiana* leaves at OD_600_ = 0.6. Combinations (*HbWRKY29* + *HbPTI5*) were co‐infiltrated in a 1:1 ratio. Leaves were photographed under white light and ultraviolet light at 48 h after infiltration. Visible cell death or necrosis indicated a HR. Three biological replicates were analysed for each treatment. Each biological replicate included three leaves.

For rubber tree, HbWRKY29‐pBin, HbPTI5‐pBin and HbTLP1‐pBin constructs were infiltrated into the abaxial side of rubber tree leaves (3rd and 4th leaves of seedlings) at OD_600_ = 0.8. After 48 h, leaves were inoculated with *E. quercicola* conidia (as above) by dusting with spores. Plants were maintained at 22°C, and disease symptoms (powdery mildew colony number and lesion size) were recorded 7 dpi. As a negative control, leaves infiltrated with pBin‐myc were similarly inoculated. Each treatment has three biological replicates. Three biological replicates were analysed for each treatment. Each biological replicate included six leaves.

### 4.14 Determination of Ionic Conductivity

The cell mortality rate was determined using the leaf disc ion leakage assay: six leaf discs (1 cm in diameter) treated with *Agrobacterium* injection were placed in 5 mL of distilled water and soaked for 5 h. The conductivity of the soaking solution was measured using a conductivity meter to obtain value A. Subsequently, the leaf discs together with the soaking solution were transferred to a sealed test tube and boiled for 20 min. After the solution cooled to room temperature, the conductivity was measured again to obtain value B. The ion leakage rate (%) was calculated as (A/B) × 100, and the experiment was repeated three times.

### 4.15 H_2_O_2_ Accumulation Detection

H_2_O_2_ accumulation was visualized by 3,3′‐diaminobenzidine (DAB) staining. Infiltrated leaves were excised 48 h post‐agroinfiltration and vacuum‐infiltrated with DAB solution (1 mg/mL, pH 3.8) for 5 min. Leaves were incubated in the dark for 6–8 h to allow brown precipitate formation. Chlorophyll was removed by boiling leaves in 95% ethanol for 10 min. Representative photographs of the stained leaves were taken for each treatment. Three biological replicates were performed for each treatment, with three leaves included in each replicate.

### 4.16 RNA Extraction and RT‐qPCR

Total RNA from plant tissues was extracted using the RNAprep Pure Polysaccharide‐Polyphenol Plant Total RNA Kit and treated with RNase‐free DNase I. First‐strand cDNA was synthesized using a PrimeScript RT kit (TaKaRa) with oligo(dT) and random primers. qPCR was performed on a CFX96 real‐time PCR system (Bio‐Rad) using SYBR Green Master Mix (TaKaRa). Each 20 μL reaction contained 2 μL of diluted cDNA and gene‐specific primers (Table S1). Thermal cycling conditions were 95°C for 3 min, followed by 40 cycles of 95°C for 10 s and 60°C for 30 s. A melt‐curve analysis confirmed single product amplification. The rubber tree *Actin* gene was used as an internal reference. Relative expression levels were calculated by the 2^−ΔΔ*C*t^ method. Three biological replicates (independent plants) with three technical replicates each were analysed.

### 4.17 Statistical Analysis

All experiments were repeated independently at least three times. Data are shown as means ± SE. Statistical significance among treatments was assessed using SPSS 22 software, via Student's *t*‐test (for two groups) or one‐way ANOVA followed by Fisher's LSD post hoc test (*p* < 0.05).

## Author Contributions

**Xiaoyu Liang:** conceptualization, writing – review and editing, writing – original draft, data curation. **Ling Xia:** software, formal analysis. **Qifeng Liu:** methodology, software. **Suna Peng:** resources. **Yiying Lu:** conceptualization, methodology, data curation, investigation. **Meng Wang:** writing – original draft, writing – review and editing, funding acquisition. **Yu Zhang:** supervision, funding acquisition, writing – original draft, writing – review and editing.

## Funding

This work was supported by the Modern Agro‐industry Technology Research System, CARS‐33‐BC1. National Natural Science Foundation of China, 32160370.

## Conflicts of Interest

The authors declare no conflicts of interest.

## Supporting information


**Figure S1:** Phylogenetic analysis of HbWRKY29 (Hb_110664140) and WRKY family proteins from *Arabidopsis*.

**Figure S2:** 3,3′‐diaminobenzidine (DAB) staining showing H_2_O_2_ accumulation upon co‐expression of *HbWRKY29* and *HbPTI5*.

**Figure S3:** Expression levels of *HbPTI5* in *HbPTI5*‐overexpressing (a) and *HbPTI5*‐silenced rubber tree plants (b).

**Figure S4:** Expression levels of *HbWRKY29* and *HbPTI5* in rubber tree plants with simultaneous gene silencing.

**Figure S5:** Sequence characteristics and bioinformatic analyses of HbTLP1.

**Figure S6:** Expression level of *HbTLP1* in different gene‐silenced rubber tree plants.

**Table S1:** The primers in this study.

**Table S2:** Gene information and accession numbers used in this study.

## Data Availability

The data that support the findings of this study are available from the corresponding author upon reasonable request.

## References

[mpp70293-bib-0001] Almagro Armenteros, J. J. , K. D. Tsirigos , C. K. Sønderby , et al. 2019. “SignalP 5.0 Improves Signal Peptide Predictions Using Deep Neural Networks.” Nature Biotechnology 37: 420–423. 10.1038/s41587-019-0036-z 30778233

[mpp70293-bib-0002] Arraño‐Salinas, P. , J. Domínguez‐Figueroa , A. Herrera‐Vásquez , et al. 2018. “WRKY7, −11 and −17 Transcription Factors Are Modulators of the bZIP28 Branch of the Unfolded Protein Response During PAMP‐Triggered Immunity in *Arabidopsis thaliana* .” Plant Science 277: 242–250. 30466590 10.1016/j.plantsci.2018.09.019

[mpp70293-bib-0003] Cao, X. , Q. Han , Y. Xiao , et al. 2024. “Population Genetic Structure of the Rubber Tree Powdery Mildew Pathogen (*Erysiphe quercicola*) From China.” Plant Disease 108: 62–70. 37467126 10.1094/PDIS-03-23-0575-RE

[mpp70293-bib-0004] Cao, Y. , F. Song , R. M. Goodman , and Z. Zheng . 2006. “Molecular Characterization of Four Rice Genes Encoding Ethylene‐Responsive Transcriptional Factors and Their Expressions in Response to Biotic and Abiotic Stress.” Journal of Plant Physiology 163: 1167–1178. 16436304 10.1016/j.jplph.2005.11.004

[mpp70293-bib-0005] Cao, Y. , Y. Wu , Z. Zheng , and F. Song . 2005. “Overexpression of the Rice EREBP‐Like Gene *OsBIERF3* Enhances Disease Resistance and Salt Tolerance in Transgenic Tobacco.” Physiological and Molecular Plant Pathology 67: 202–211.

[mpp70293-bib-0006] Cui, Z. , F. Liang , J. Zhang , F. Wang , D. Liu , and H. Wang . 2021. “Transgenic Expression of *TaTLP1*, a Thaumatin‐Like Protein Gene, Reduces Susceptibility to Common Root Rot and Leaf Rust in Wheat.” Crop Journal 9: 1214–1218.

[mpp70293-bib-0007] Dang, F. , J. Lin , Y. Li , et al. 2023. “SlWRKY30 and SlWRKY81 Synergistically Modulate Tomato Immunity to *Ralstonia solanacearum* by Directly Regulating *SlPR‐STH2* .” Horticulture Research 10: uhad052. 37206055 10.1093/hr/uhad050 PMC10189802

[mpp70293-bib-0008] de Jesús‐Pires, C. , J. R. Ferreira‐Neto , J. Pacifico Bezerra‐Neto , et al. 2020. “Plant Thaumatin‐Like Proteins: Function, Evolution and Biotechnological Applications.” Current Protein and Peptide Science 21: 36–51. 30887921 10.2174/1389203720666190318164905

[mpp70293-bib-0010] Gu, Y. Q. , and G. B. Martin . 1998. “Molecular Mechanisms Involved in Bacterial Speck Disease Resistance of Tomato.” Philosophical Transactions of the Royal Society, B: Biological Sciences 353: 1455–1461.

[mpp70293-bib-0011] Gu, Y. Q. , M. C. Wildermuth , S. Chakravarthy , et al. 2002. “Tomato Transcription Factors Pti4, Pti5, and Pti6 Activate Defense Responses When Expressed in *Arabidopsis* .” Plant Cell 14: 817–831. 11971137 10.1105/tpc.000794 PMC150684

[mpp70293-bib-0012] Gu, Y. Q. , C. Yang , V. K. Thara , J. Zhou , and G. B. Martin . 2000. “ *Pti4* Is Induced by Ethylene and Salicylic Acid, and Its Product Is Phosphorylated by the Pto Kinase.” Plant Cell 12: 771–786. 10810149 10.1105/tpc.12.5.771 PMC139926

[mpp70293-bib-0013] Hallgren, J. , K. D. Tsirigos , M. D. Pedersen , et al. 2022. “DeepTMHMM Predicts Alpha and Beta Transmembrane Proteins Using Deep Neural Networks.” bioRxiv 8: 487609. [Preprint]

[mpp70293-bib-0014] Hao, Z. , J. Tian , H. Fang , et al. 2022. “A VQ‐Motif‐Containing Protein Fine‐Tunes Rice Immunity and Growth by a Hierarchical Regulatory Mechanism.” Cell Reports 40: 111235. 35977497 10.1016/j.celrep.2022.111235

[mpp70293-bib-0015] Huang, H. , W. Zhao , C. Li , et al. 2022. “SlVQ15 Interacts With Jasmonate‐ZIM Domain Proteins and SlWRKY31 to Regulate Defense Response in Tomato.” Plant Physiology 190: 828–842. 35689622 10.1093/plphys/kiac275 PMC9434178

[mpp70293-bib-0016] Huang, S. Q. , J. Q. Zhang , Y. Zhu , et al. 2024. “Revealing the Structure‐Property Difference of Natural Rubber Prepared by Different Methods: Protein and Gel Content Are Key Factors.” Chinese Journal of Polymer Science 42: 457–467.

[mpp70293-bib-0017] Kyte, J. , and R. F. Doolittle . 1982. “A Simple Method for Displaying the Hydropathic Character of a Protein.” Journal of Molecular Biology 157: 105–132. 7108955 10.1016/0022-2836(82)90515-0

[mpp70293-bib-0018] Lan, L. , L. Cao , L. Zhang , et al. 2025. “A Novel Mode of WRKY1 Regulating PR1‐Mediated Immune Balance to Defend Against Powdery Mildew in Apple.” Molecular Horticulture 5: 17. 40038814 10.1186/s43897-024-00141-z PMC11881497

[mpp70293-bib-0019] Li, Q. , B. Xian , Q. Yu , et al. 2024. “The CsAP2‐09‐CsWRKY25‐CsRBOH2 Cascade Confers Resistance Against Citrus Bacterial Canker by Regulating ROS Homeostasis.” Plant Journal 118: 534–548. 10.1111/tpj.16623 38230828

[mpp70293-bib-0020] Liang, X. , Z. Ma , Y. Ke , et al. 2023. “Single‐Cell Transcriptomic Analyses Reveal Cellular and Molecular Patterns of Rubber Tree Response to Early Powdery Mildew Infection.” Plant, Cell & Environment 46: 2222–2237. 10.1111/pce.14585 36929646

[mpp70293-bib-0021] Liang, X. , Y. Zhang , S. Wan , et al. 2025. “Impacts of Sulfur Application on Microbial Communities and Functional Attributes in Rubber Plantation Soil.” BMC Microbiology 25: 265. 40316901 10.1186/s12866-025-03971-z PMC12046827

[mpp70293-bib-0022] Lin, R. , W. Zhao , X. Meng , and Y. Peng . 2007. “Molecular Cloning and Characterization of a Rice Gene Encoding AP2/EREBP‐Type Transcription Factor and Its Expression in Response to Infection With Blast Fungus and Abiotic Stresses.” Physiological and Molecular Plant Pathology 70: 60–68.

[mpp70293-bib-0023] Liu, Q. , A. Qiao , S. Zhou , et al. 2024. “Identification of the *HbZAR1* Gene and Its Potential Role as a Minor Gene in Response to Powdery Mildew and Anthracnose of *Hevea brasiliensis* .” Forests 15: 1891.

[mpp70293-bib-0024] Liu, Y. , Q. Liu , X. Li , et al. 2023. “MdERF114 Enhances the Resistance of Apple Roots to *Fusarium solani* by Regulating the Transcription of *MdPRX63* .” Plant Physiology 192: 2015–2029. 36721923 10.1093/plphys/kiad057 PMC10315273

[mpp70293-bib-0025] Liyanage, K. K. , S. Khan , P. E. Mortimer , et al. 2016. “Powdery Mildew Disease of Rubber Tree.” Forest Pathology 46: 90–103.

[mpp70293-bib-0043] Mu, Y. , Y. Dong , X. Li , et al. 2024. “JrPHL8‐JrWRKY4‐JrSTH2L Module Regulates Resistance to *Colletotrichum gloeosporioides* in Walnut.” Horticulture Research 11: uhae148. 38988616 10.1093/hr/uhae148 PMC11233879

[mpp70293-bib-0026] Ohme‐Takagi, M. , and H. Shinshi . 1995. “Ethylene‐Inducible DNA Binding Proteins That Interact With an Ethylene‐Responsive Element.” Plant Cell 7: 173–182. 7756828 10.1105/tpc.7.2.173 PMC160773

[mpp70293-bib-0027] Oñate‐Sánchez, L. , J. P. Anderson , J. Young , and K. B. Singh . 2007. “AtERF14, a Member of the ERF Family of Transcription Factors, Plays a Nonredundant Role in Plant Defense.” Plant Physiology 143: 400–409. 17114278 10.1104/pp.106.086637 PMC1761963

[mpp70293-bib-0028] Pandey, S. P. , M. Roccaro , M. Schön , E. Logemann , and I. E. Somssich . 2010. “Transcriptional Reprogramming Regulated by WRKY18 and WRKY40 Facilitates Powdery Mildew Infection of *Arabidopsis* .” Plant Journal 64: 912–923. 10.1111/j.1365-313X.2010.04387.x 21143673

[mpp70293-bib-0029] Rushton, P. J. , I. E. Somssich , P. Ringler , and Q. J. Shen . 2010. “WRKY Transcription Factors.” Trends in Plant Science 15: 247–258. 20304701 10.1016/j.tplants.2010.02.006

[mpp70293-bib-0030] van Loon, L. C. , M. Rep , and C. M. J. Pieterse . 2006. “Significance of Inducible Defense‐Related Proteins in Infected Plants.” Annual Review of Phytopathology 44: 135–162. 10.1146/annurev.phyto.44.070505.143425 16602946

[mpp70293-bib-0031] Wang, F. , S. Yuan , W. Wu , et al. 2020. “TaTLP1 Interacts With TaPR1 to Contribute to Wheat Defense Responses to Leaf Rust Fungus.” PLoS Genetics 16: e1008713. 32658889 10.1371/journal.pgen.1008713 PMC7357741

[mpp70293-bib-0032] Wang, M. , H. Xiao , X. Li , et al. 2023. “Functional Characterization of Powdery Mildew Resistance‐Related Genes *HbSGT1a* and *HbSGT1b* in *Hevea brasiliensis* Muell. Arg.” European Journal of Plant Pathology 165: 153–161.

[mpp70293-bib-0033] Wang, Y. , X. Wang , J. Fang , et al. 2023. “VqWRKY56 Interacts With VqbZIPC22 in Grapevine to Promote Proanthocyanidin Biosynthesis and Increase Resistance to Powdery Mildew.” New Phytologist 237: 1856–1875. 36527243 10.1111/nph.18688

[mpp70293-bib-0034] Yan, C. , W. Liu , R. Li , G. Liu , and Y. Wang . 2025. “VqERF1B‐VqERF062‐VqNSTS2 Transcriptional Cascade Enhances Stilbene Biosynthesis and Resistance to Powdery Mildew in Grapevine.” Plant Biotechnology Journal 23: 2065–2082. 40062824 10.1111/pbi.70041 PMC12120875

[mpp70293-bib-0035] Yang, Y. , F. Zhao , H. Zhang , and S. Liao . 2025. “Role of the Controlled Fluorinated Polymer Side Chains Fabricating via ARGET‐ATRP on Natural Rubber Molecular Network.” International Journal of Biological Macromolecules 322: 147000. 40840742 10.1016/j.ijbiomac.2025.147000

[mpp70293-bib-0036] Yin, W. , X. Wang , H. Liu , et al. 2022. “Overexpression of VqWRKY31 Enhances Powdery Mildew Resistance in Grapevine by Promoting Salicylic Acid Signaling and Specific Metabolite Synthesis.” Horticulture Research 9: uhab064. 35043152 10.1093/hr/uhab064 PMC8944305

[mpp70293-bib-0037] Zhai, D. L. , P. Thaler , F. R. Worthy , and J. Xu . 2023. “Rubber Latex Yield Is Affected by Interactions Between Antecedent Temperature, Rubber Phenology, and Powdery Mildew Disease.” International Journal of Biometeorology 67: 1569–1579. 37522973 10.1007/s00484-023-02515-2

[mpp70293-bib-0038] Zhang, X. , Q. Zhang , Y. Zhu , et al. 2025. “A Module With Multiple Transcription Factors Positively Regulates Powdery Mildew Resistance in Grapevine.” Plant Biotechnology Journal 23: 3984–3999. 40534152 10.1111/pbi.70196 PMC12392958

[mpp70293-bib-0039] Zhou, J. , Y. T. Loh , R. A. Bressan , and G. B. Martin . 1995. “The Tomato Gene *Pti1* Encodes a Serine/Threonine Kinase That Is Phosphorylated by Pto and Is Involved in the Hypersensitive Response.” Cell 83: 925–935. 8521516 10.1016/0092-8674(95)90208-2

[mpp70293-bib-0040] Zhou, J. , X. Tang , and G. B. Martin . 1997. “The Pto Kinase Conferring Resistance to Tomato Bacterial Speck Disease Interacts With Proteins That Bind a *Cis*‐Element of Pathogenesis‐Related Genes.” EMBO Journal 16: 3207–3218. 9214637 10.1093/emboj/16.11.3207 PMC1169938

[mpp70293-bib-0041] Zhou, M. , H. Wang , X. Yu , et al. 2024. “Transcription Factors VviWRKY10 and VviWRKY30 co‐Regulate Powdery Mildew Resistance in Grapevine.” Plant Physiology 195: 446–461. 38366578 10.1093/plphys/kiae080

[mpp70293-bib-0042] Zhou, R. , Y. Dong , X. Liu , et al. 2022. “JrWRKY21 Interacts With JrPTI5L to Activate the Expression of *JrPR5L* for Resistance to *Colletotrichum gloeosporioides* in Walnut.” Plant Journal 111: 1152–1166. 10.1111/tpj.15883 35765867

