# Supplementary figures and images for: The Transcription Factors HbWRKY29 and HbPTI5 cooperatively enhance rubber tree resistance to powdery mildew
Source: Mol Plant Pathol. 2026 Jun 11;27(6):e70293. doi: 10.1111/mpp.70293 (PMC13260869; doi:10.1111/mpp.70293)

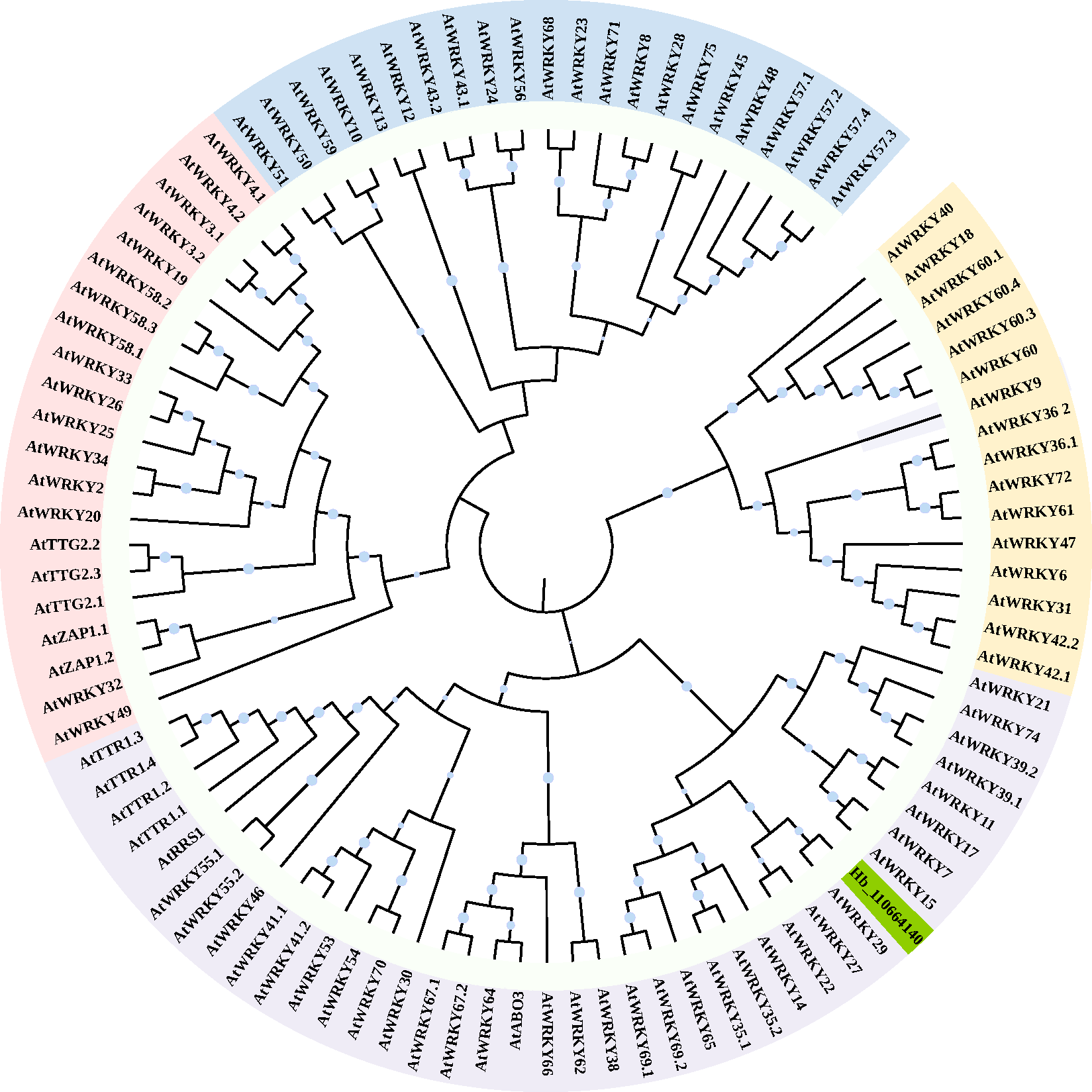


**Figure S1 Phylogenetic analysis of HbWRKY29 (Hb_110664140) and WRKY family proteins from *Arabidopsis***.

Supplement: Supplementary file 1 — Figure S1: Phylogenetic analysis of HbWRKY29 (Hb_110664140) and WRKY family proteins from Arabidopsis. [file MPP-27-e70293-s005.docx]

**
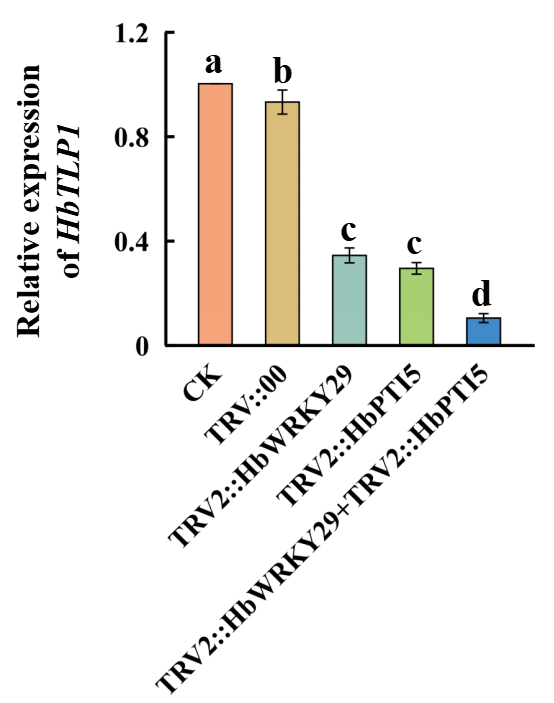
**

**Figure S6 Expression level of *HbTLP1* in different gene-silenced rubber tree plants.**

Supplement: Supplementary file 6 — Figure S6: Expression level of HbTLP1 in different gene‐silenced rubber tree plants. [file MPP-27-e70293-s003.docx]
