# Supplementary material for: The Transcription Factors HbWRKY29 and HbPTI5 cooperatively enhance rubber tree resistance to powdery mildew
Source: Mol Plant Pathol. 2026 Jun 11;27(6):e70293. doi: 10.1111/mpp.70293 (PMC13260869; doi:10.1111/mpp.70293)
Supplement: Supplementary file 2 — Figure S2: 3,3′‐diaminobenzidine (DAB) staining showing H2O2 accumulation upon co‐expression of HbWRKY29 and HbPTI5. [file MPP-27-e70293-s008.docx]

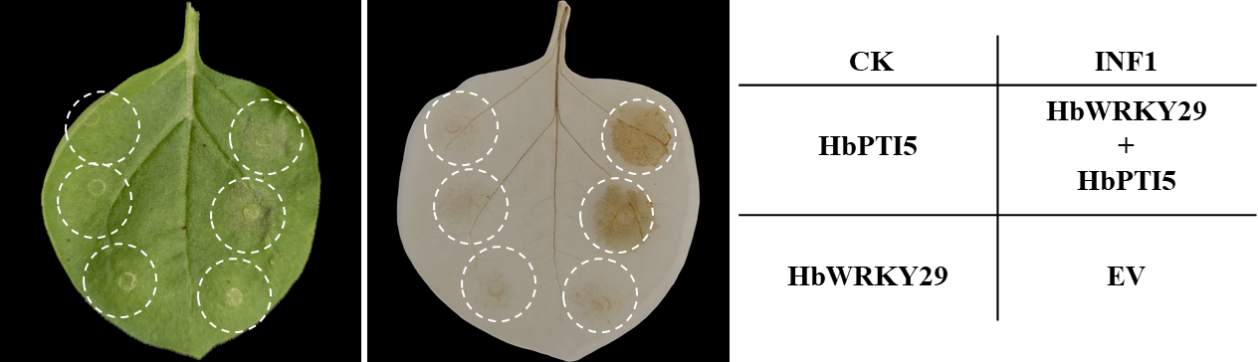


**Figure S2 DAB staining showing H₂O₂ accumulation upon co-expression of *HbWRKY29* and *HbPTI5*.** Each label on the right corresponds to the DAB signal observed at the indicated position in the image.
