# Supplementary material for: The Transcription Factors HbWRKY29 and HbPTI5 cooperatively enhance rubber tree resistance to powdery mildew
Source: Mol Plant Pathol. 2026 Jun 11;27(6):e70293. doi: 10.1111/mpp.70293 (PMC13260869; doi:10.1111/mpp.70293)
Supplement: Supplementary file 3 — Figure S3: Expression levels of HbPTI5 in HbPTI5‐overexpressing (a) and HbPTI5‐silenced rubber tree plants (b). [file MPP-27-e70293-s004.docx]

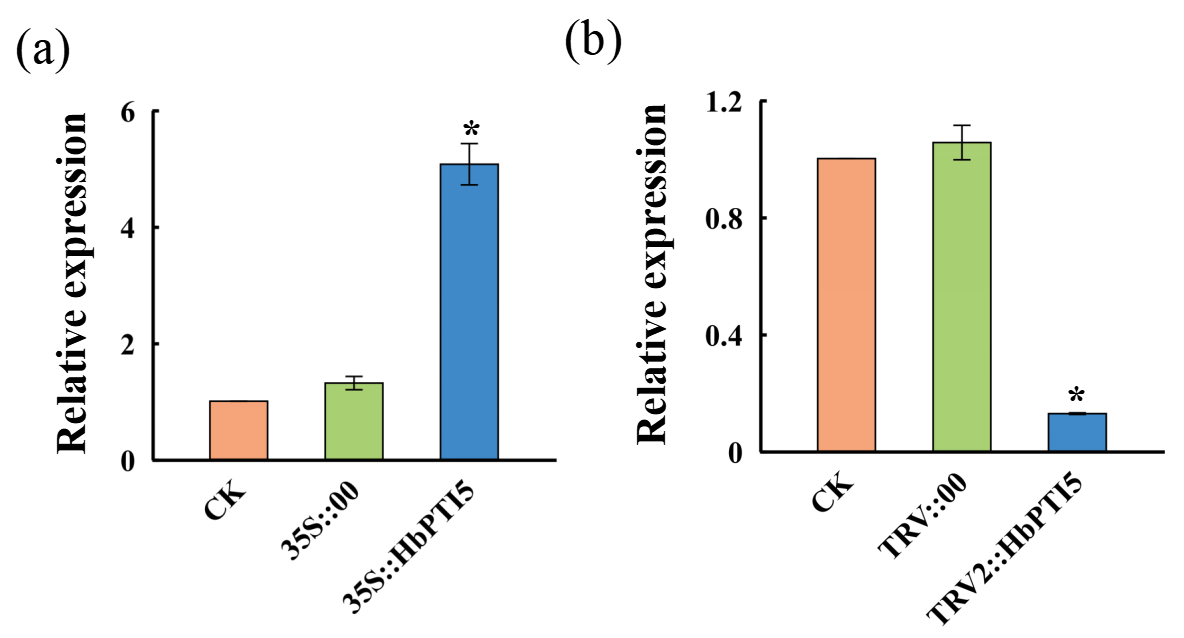


**Figure S3 Expression levels of *HbPTI5* in *HbPTI5*-overexpressing (a) and *HbPTI5*-silenced rubber tree plants (b).**
