# Supplementary material for: The Transcription Factors HbWRKY29 and HbPTI5 cooperatively enhance rubber tree resistance to powdery mildew
Source: Mol Plant Pathol. 2026 Jun 11;27(6):e70293. doi: 10.1111/mpp.70293 (PMC13260869; doi:10.1111/mpp.70293)
Supplement: Supplementary file 4 — Figure S4: Expression levels of HbWRKY29 and HbPTI5 in rubber tree plants with simultaneous gene silencing. [file MPP-27-e70293-s002.docx]

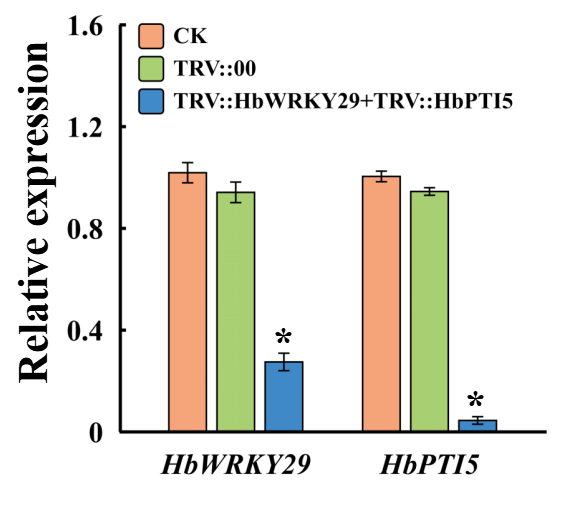


**Figure S4 Expression levels of *HbWRKY29* and *HbPTI5* in rubber tree plants with simultaneous gene silencing.**
