# Supplementary material for: The Transcription Factors HbWRKY29 and HbPTI5 cooperatively enhance rubber tree resistance to powdery mildew
Source: Mol Plant Pathol. 2026 Jun 11;27(6):e70293. doi: 10.1111/mpp.70293 (PMC13260869; doi:10.1111/mpp.70293)
Supplement: Supplementary file 5 — Figure S5: Sequence characteristics and bioinformatic analyses of HbTLP1. [file MPP-27-e70293-s001.docx]

**
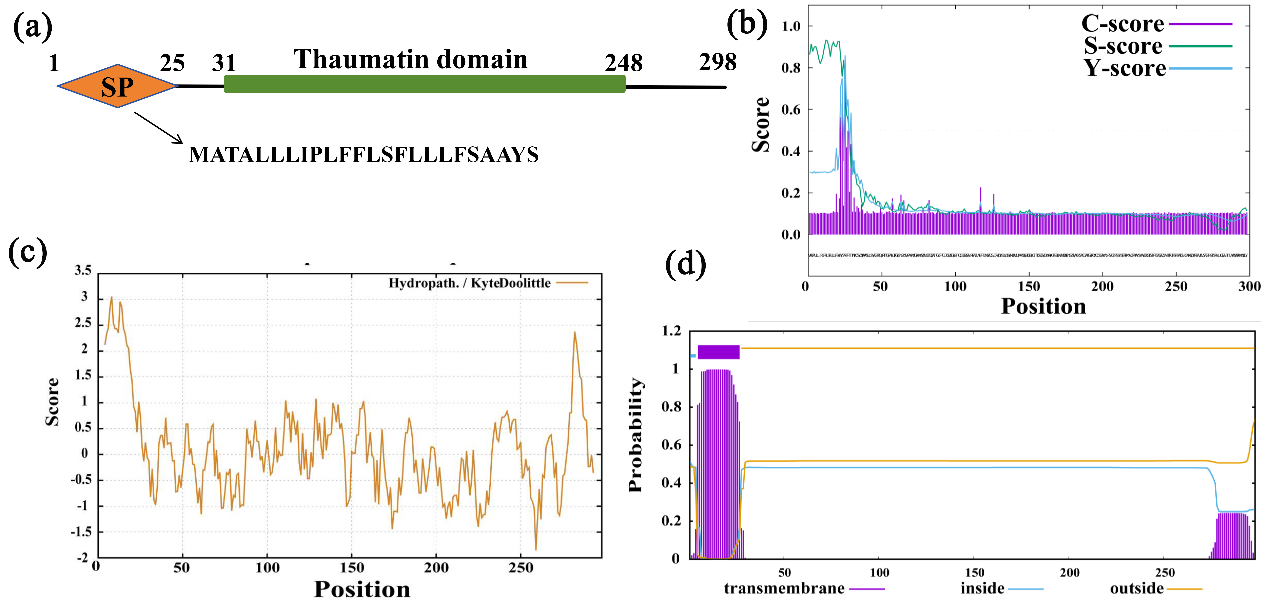
**

**Figure S5 Sequence characteristics and bioinformatic analyses of HbTLP1.** (a) Schematic diagram of the HbTLP1 protein structure showing the N-terminal signal peptide (SP) and conserved thaumatin domain. The predicted signal peptide sequence is shown below. (b) Signal peptide prediction of HbTLP1 using SignalP. C-score, S-score, and Y-score indicate the predicted signal peptide cleavage probability and signal peptide characteristics. (c) Hydrophobicity analysis of HbTLP1 based on the Kyte–Doolittle algorithm. (d) Predicted transmembrane topology of HbTLP1. The purple region indicates the predicted transmembrane segment, while the blue and orange lines represent intracellular and extracellular localization probabilities, respectively.
