# Supplementary material for: The Transcription Factors HbWRKY29 and HbPTI5 cooperatively enhance rubber tree resistance to powdery mildew
Source: Mol Plant Pathol. 2026 Jun 11;27(6):e70293. doi: 10.1111/mpp.70293 (PMC13260869; doi:10.1111/mpp.70293)
Supplement: Supplementary file 7 — Table S1: The primers in this study. [file MPP-27-e70293-s007.docx]

**Table S1 The primers in this study**

| **Name** | **Sequence (5' to 3')** |
| --- | --- |
| HbWRKY29-QF | GATGATGCTTCAGTGGCAGC |
| HbWRKY29-QR | CCTTGATGGGTTTCTGCCCA |
| HbPTI5-QF | GAAATTCGCGACTCCACACG |
| HbPTI5-QR | CACGCATACTAAAAGCCGCC |
| HbTLP1-QF | GCAAGCCCAGCTCTTACTCT |
| HbTLP1-QR | CCGATTTCAGACCGGTAGCA |
| HbACTIN-QF | GATGTGGATATCAGGAAGGA |
| HbACTIN-QR | CATACTGCTTGGAGCAAGA |
| HbWRKY29-F | ATGGAAGACTGGGATCTGCGA |
| HbWRKY29-R | AACAACAGTAGCTGCTGAATGATGA |
| HbPTI5-F | ATGGAGAATCGAAAACACATGGT |
| HbPTI5-R | ACAAACTCTAAGCAAATGATCAGAACTC |
| HbTLP1-F | ATGGCCACTGCTCTGCTACTCA |
| HbTLP1-R | ATACAGCAGCTGCCACAACCG |
| HbWRKY29-GFP-F | gagaacacgggggactctagaATGGAAGACTGGGATCTGCGA |
| HbWRKY29-GFP-R | gctcaccatggatccggtaccAACAACAGTAGCTGCTGAATGATGA |
| HbPTI5-GFP-F | gagaacacgggggactctagaATGGAGAATCGAAAACACATGGT |
| HbPTI5-GFP-R | gctcaccatggatccggtaccACAAACTCTAAGCAAATGATCAGAACTC |
| HbTLP1-GFP-F | gagaacacgggggactctagaATGGCCACTGCTCTGCTACTCA |
| HbTLP1-GFP-R | gctcaccatggatccggtaccATACAGCAGCTGCCACAACCG |
| 35S-GFP-F | cactatccttcgcaagaccct |
| 35S-GFP-R | ttcagggtcagcttgccgtag |
| HbWRKY29-pGBKT7-F | atggccatggaggccgaattcATGGAAGACTGGGATCTGCGA |
|  |  |
| HbWRKY29-pGBKT7-R | atgcggccgctgcaggtcgacCTAAACAACAGTAGCTGCTGAATGATG |
| HbPTI5-pGADT7-F | gccatggaggccagtgaattcATGGAGAATCGAAAACACATGGTTCT |
| HbPTI5-pGADT7-R | cagctcgagctcgatggatccTCAACAAACTCTAAGCAAATGATCAGAACTCAC |
| HbTLP1-pGBKT7-F | AtggccatggaggccgaattcATGGCCACTGCTCTGCTACTCA |
| HbTLP1-pGBKT7-R | atgcggccgctgcaggtcgacTCAATACAGCAGCTGCCACAA |
| pGBKT7-F | taatacgactcactatagggc |
| pGBKT7-R | ttttcgttttaaaacctaagagtc |
| pGADT7-F | aataccactacaatggatgatg |
| pGADT7-R | gagatggtgcacgatgcacagt |
| HbWRKY29-eYFPN-F | tggcgcgccactagtggatccATGGAAGACTGGGATCTGCGA |
|  |  |
| HbWRKY29-eYFPN-R | ctccatcccgggagcggtaccAACAACAGTAGCTGCTGAATGATG |
| HbPTI5-eYFPN-F | tggcgcgccactagtggatccATGGAGAATCGAAAACACATGGT |
| HbPTI5-eYFPN-R | ctccatcccgggagcggtaccTCAACAAACTCTAAGCAAATGATCAG |
| HbTLP1-eYFPN-F | tggcgcgccactagtggatccATGGAGAATCGAAAACACATGGT |
| HbTLP1-eYFPN-R | ctccatcccgggagcggtaccTCAACAAACTCTAAGCAAATGATCAG |
| HbPTI5-eYFPC-F | tggcgcgccactagtggatccATGGAGAATCGAAAACACATGGT |
| HbPTI5-eYFPC-R | gtacatcccgggagcggtaccACAAACTCTAAGCAAATGATCAGACTC |
| HbWRKY29-cLUC-F | TACGCGTCCCGGGGC GGTACCATGGGTGAGTTTGCTTTCATGG |
| HbWRKY29-cLUC-R | ACGAAAGCTCTGCAGGTCGACAGTAAAATCAATTTCCCTAGGAAACAA |
| HbPTI5-cLUC-F | TACGCGTCCCGGGGCGGTACCATGGAGAATCGAAAACACATGGT |
| HbPTI5-cLUC-R | ACGAAAGCTCTGCAGGTCGACACAAACTCTAAGCAAATGATCAGAACTC |
| HbWRKY29-nLUC-F | ACGGGGGACGAGCTCGGTACCATGGAAGACTGGGATCTGCGA |
| HbWRKY29-nLUC-R | CGCGTACGAGATCTGGTCGACAACAACAGTAGCTGCTGAATGATG |
| HbPTI5-nLUC-F | ACGGGGGACGAGCTCGGTACCATGGAGAATCGAAAACACATGGT |
| HbPTI5-nLUC-R | CGCGTACGAGATCTGGTCGACACAAACTCTAAGCAAATGATCAGAACTC |
| HbTLP1-nLUC-F | ACGGGGGACGAGCTCGGTACCATGGCCACTGCTCTGCTACTCA |
| HbTLP1-nLUC-R | CGCGTACGAGATCTGGTCGACATACAGCAGCTGCCACAACCG |
| Nluc-R | TCTCTTCATAGCCTTATGCA |
| Cluc-F | GTTGCGCGGAGGAGTTGTGT |
| Cluc-R | ACATGTTTCCCAATGCCATA |
| HbWRKY29-pBin-F | aattctgcagtcgaccccgggATGGAAGACTGGGATCTGCGA |
| HbWRKY29-pBin-R | caagaaagctgggtccccgggAACAACAGTAGCTGCTGAATGATG |
| HbPTI5-pBin-F | aattctgcagtcgaccccgggATGGAGAATCGAAAACACATGGT |
| HbPTI5-pBin-R | caagaaagctgggtccccgggACAAACTCTAAGCAAATGATCAG |
| HbTLP1-pBin-F | aattctgcagtcgaccccgggATGGCCACTGCTCTGCTACTCA |
| HbTLP1-pBin-R | caagaaagctgggtccccgggATACAGCAGCTGCCACAACCG |
| pBin-35S-myc-F | GAATCTCAAGCAATCAAGCA |
| pBin-35S-myc-R | GGTTGATAACAGCGGGTTAA |
| HbPTI5-qdz-F | CACGTGGCACCATGAGATGA |
| HbPTI5-qdz-R | GCCGCCCTATCATAAGCCAA |
| HbTLP1-qdz-F | TCTTGTAGCCGGTCTCCTGT |
| HbTLP1-qdz-R | GCCAAACTTGGTAGCTGCAC |
| T7promoter-F | taatacgactcactataggg |
| phis-R | ctcaacgattagcgacc |
| HbPTI5-phis-F | gactcactatagggcgaattcAAATGCTACAGTGCATCAAGAGGTTAG |
| HbPTI5-phis-R | gatcgattcgcgaacgcgtgagctcGGCCTGGAGGAAGACTAAAGAATGAAG |
| HbTLP1-phis-F | gactcactatagggcgaattcTTGCTCTCTCTTCCCGTGTCG |
| HbTLP1-phis-R | gatcgattcgcgaacgcgtgagctcTGGAGAATTTAGCGCGAGTGTTGG |
| 62KS-F | CGCAAGACCCTTCCTCTAT |
| 62KS-R | CGGGAAACTACTCACACATTA |
| HbPTI5-62KS-F | cgctctagaactagtggatcc‌ATGGAGAATCGAAAACACATGGTTCT |
| HbPTI5-62KS-R | gtcgacggtatcgataagcttTCAACAAACTCTAAGCAAATGATCAGAACTCAC |
| HbWRKY29-62KS-F | CGCTCTAGAACTAGTGGATCCatggaagactgggatctgcg |
| HbWRKY29-62KS-R | GTCGACGGTATCGATAAGCTTctaaacaacagtagctgctgaatgatgatc |
| PG0800-F | gtgctgcaaggcgattaagt |
| PG0800-R | agggcgtatctcttcatagcc |
| HbPTI5-PG0800-F | gggccccccctcgaggtcgacAAATGCTACAGTGCATCAAGAGGTTAG |
| HbPTI5-PG0800-R | cgctctagaactagtggatccGGCCTGGAGGAAGACTAAAGAATGAAG |
| TLP-PG0800-F | gggccccccctcgaggtcgacTTGCTCTCTCTTCCCGTGTCG |
| TLP-PG0800-R | cgctctagaactagtggatccTGGAGAATTTAGCGCGAGTGTTGG |
| TRV2-F | acggacgagtggacttagat |
| TRV2-R | attgaacctaaaacttcagac |
| HbWRKY29-TRV2-F | agaaggcctccatggggatcATGGAAGACTGGGATCTGCG |
| HbWRKY29-TRV2-R | cctcgagacgcgtgagctcgGTGTTTCTTCTGCAGTTTCTCTGGC |
| HbPTI5-TRV2-F | agaaggcctccatggggatcATGGAGAATCGAAAACACATGGTTCT |
| HbPTI5-TRV2-R | cctcgagacgcgtgagctcgCTCCGCCGTCTCGAACG |
| HbTLP1-TRV2-F1 | agaaggcctccatggggatcTTGTTTTCAGCTGCATATTCGGCT |
| HbTLP1-TRV2-R1 | cctcgagacgcgtgagctcgTTCTGCAAGGGTGGCAGGAG |
