# Supplementary material for: The Transcription Factors HbWRKY29 and HbPTI5 cooperatively enhance rubber tree resistance to powdery mildew
Source: Mol Plant Pathol. 2026 Jun 11;27(6):e70293. doi: 10.1111/mpp.70293 (PMC13260869; doi:10.1111/mpp.70293)
Supplement: Supplementary file 8 — Table S2: Gene information and accession numbers used in this study. [file MPP-27-e70293-s006.docx]

**Table S2 Gene information and accession numbers used in this study**

| **Name** | **Genus and species** | **Login number** |
| --- | --- | --- |
| *HbWRKY29* | *Hevea brasiliensis* | XP_057994678.1 |
| *HbPTI5* | *Hevea brasiliensis* | XP_021692420.2 |
| *HbTLP1* | *Hevea brasiliensis* | XP_021650711.2 |
| *MeWRKY29* | *Manihot esculenta* | XP_021629178.1 |
| *JrWRKY29* | *Juglans regia* | XP_018811192.1 |
| *JcWRKY29* | *Jatropha curcas* | XP_020539527.1 |
| *RcWRKY29* | *Ricinus communis* | XP_025013358.1 |
| *MiWRKY29* | *Mangifera indica* | XP_044485443.1 |
| *SsWRKY29* | *Salix suchowensis* | KAJ6347391.1 |
| *DzWRKY29* | *Durio zibethinus* | XP_022737617.1 |
| *PnWRKY29* | *Populus nigra* | KAI5601380.1 |
| *AtWRKY29* | *Arabidopsis thaliana* | NP_194086.4 |
| *MePTI5* | *Manihot esculenta* | XP_021598613.1 |
| *JrPTI5* | *Juglans regia* | XP_041028885.1 |
| *JcPTI5* | *Jatropha curcas* | XP_012091701.1 |
| *RcPTI5* | *Ricinus communis* | XP_002522517 |
| *MiPTI5* | *Macadamia integrifolia* | XP_042478165.1 |
| *SsPTI5* | *Spatholobus suberectus* | TKY45337.1 |
| *DzPTI5* | *Durio zibethinus* | XP_022723886.1 |
| *CaPTI5* | *Coffea arabica* | XP_027111850.1 |
| *HsPTI5* | *Hibiscus syriacus* | XP_039016644.1 |
| *FcTLP1* | *Fagus crenata* | GMY12239.1 |
| *Md**TLP1* | *Malus domestica* | XP_008337997.3 |
| *MnTLP1* | *Morus notabilis* | XP_024032884.1 |
| *SsTLP1* | *Stylosanthes scabra* | MED6108446.1 |
| *TrTLP1* | *Trifolium repens* | WJX55753.1 |
| *ApTLP1* | *Abrus precatorius* | XP_027362420.1 |
| *CcTLP1* | *Cajanus cajan* | XP_020216368.2 |
| *HsTLP1* | *Hibiscus syriacus* | KAE8709497.1 |
| *MeTLP1* | *Manihot esculenta* | XP_043810031.1 |
| *ElTLP1* | *Euphorbia lathyris* | XP_065855171.1 |
| *JcTLP1* | *Jatropha curcas* | XP_012065985.1 |
| *PnTLP1* | *Populus nigra* | XP_061971553.1 |
| *PvTLP1* | *Pistacia vera* | XP_031258510.1 |
| *MiTLP1* | *Mangifera indica* | XP_044492344.1 |
| *NbTLP1* | *Nicotiana benthamiana* | NP_001311972.1 |
| *JrTLP1* | *Juglans regia* | XP_018849979.1 |
